# Orphan lysosomal solute carrier MFSD1 facilitates highly selective dipeptide transport

**DOI:** 10.1073/pnas.2319686121

**Published:** 2024-03-20

**Authors:** Danila Boytsov, Gregor M. Madej, Georg Horn, Nadine Blaha, Thomas Köcher, Harald H. Sitte, Daria Siekhaus, Christine Ziegler, Walter Sandtner, Marko Roblek

**Affiliations:** ^a^Institute of Pharmacology, Center for Physiology and Pharmacology, Medical University of Vienna, Vienna AT-1090, Austria; ^b^Department of Biophysics II/Structural Biology, University of Regensburg, Regensburg DE-93053, Germany; ^c^Vienna BioCenter Core Facilities, Metabolomics, Vienna BioCenter, Vienna AT-1030, Austria; ^d^Hourani Center for Applied Scientific Research, Al-Ahliyya Amman University, Amman JO-19328, Jordan; ^e^Center for Addiction Research and Science, Medical University of Vienna, Vienna AT-1090, Austria; ^f^Institute of Science and Technology Austria, Klosterneuburg AT-3400, Austria

**Keywords:** deorphanization of SLC MFSD1, targeted metabolomics, electrophysiology, dipeptides, lysosomes

## Abstract

Orphan solute carrier (SLC) represents a group of membrane transporters whose exact functions and substrate specificities are not known. Elucidating the function and regulation of orphan SLC transporters is not only crucial for advancing our knowledge of cellular and molecular biology but can potentially lead to the development of new therapeutic strategies. Here, we provide evidence for the biological function of a ubiquitous orphan lysosomal SLC, the Major Facilitator Superfamily Domain-containing Protein 1 (MFSD1), which has remained phylogenetically unassigned. Targeted metabolomics revealed that dipeptides containing either lysine or arginine residues accumulate in lysosomes of cells lacking MFSD1. Whole-cell patch-clamp electrophysiological recordings of HEK293-cells expressing MFSD1 on the cell surface displayed transport affinities for positively charged dipeptides in the lower mM range, while dipeptides that carry a negative net charge were not transported. This was also true for single amino acids and tripeptides, which MFSD1 failed to transport. Our results identify MFSD1 as a highly selective lysosomal lysine/arginine/histidine-containing dipeptide exporter, which functions as a uniporter.

## Significance

The proper distribution of metabolites between the cell interior and exterior, as well as within cellular subcompartments, is of the utmost importance for homeostasis. Proteins of the solute carrier family (SLC) facilitate the movement of metabolites through cellular membranes. Lysosomes are organelles that degrade biological material. The degradation products are transported by lysosomal SLCs into the cytoplasm to allow for their reuse. Here we show that the ubiquitous SLC Major Facilitator Superfamily Domain-containing Protein 1 (MFSD1) acts as a lysosomal dipeptide exporter. The absence of functional MFSD1 is known to cause immune and liver malfunction. Our study lays the foundation for dissecting how altered levels of lysosomal and cytosolic dipeptides give rise to disease.

Cellular homeostasis and metabolic pathways rely on the proper distribution of metabolites which is ensured by specific controlled metabolite transport across biological membranes. Solute carrier (SLC) transporters are a class of membrane proteins that play crucial roles in the transport of metabolites, such as amino acids, sugars, nucleotides, metals, vitamins, neurotransmitters, and ions. They are driven by (electro)chemical gradients and malfunctioning SLCs are associated with a variety of metabolic diseases (1, 2).

SLCs are assigned according to their respective substrate spectrum, transport mechanism, and lately also by their structural fold to 66 different SLC families (3–5). Despite the biological and clinical impact, the substrate specificity and transport mechanism of approximately 30% of mammalian carriers remain unknown as they show low sequence similarities and distinct structural features when compared to well-studied SLC transporters (4, 6). Being mainly identified through genetic and genomic studies, the specific functions, the substrate spectrum, and the transport mechanism of these orphan SLCs have not been fully characterized. Therefore, understanding the exact biological roles of orphan SLC transporters remains challenging.

Recently, we have demonstrated the involvement of the orphan lysosomal SLC Major Facilitator Superfamily Domain-containing Protein 1 (MFSD1) in regulating cell migration (7). MFSD1 increases the amount of inactive β1 integrins recycled back to the cell surface and thereby lowers the ratio of active to inactive integrin, i.e., the integrin activation index. Cells lacking MFSD1 showed faster migration and increased turnover of focal adhesions, which can contribute to metastasis (7). The loss of MFSD1 leads to alterations in the metabolite environment within the endo-lysosomal system, affecting the proper functioning of proteins involved in inactive β1 integrin recycling. Therefore, it is an intriguing question how MFSD1 can so efficiently and selectively affect the complex process of integrin recycling.

MFSD1 is predicted to adopt the Major Facilitator Superfamily MFS-fold, which represents the most frequent structural fold of secondary active transporters, being found in 16 of the 66 SLC families (8). Despite their highly conserved structural fold, MFS transporters share very low sequence identity and substrate specificity can rarely be deduced from sequence or structural alignments. A phylogenic Bayesian-based study related MFSD1 to SLC29 nucleoside transporters (9), while a recent study on structure and evolutionary-based classification of SLCs using the AlphaFold model related MFSD1 to monocarboxylate (SLC16) and sugar-phosphate (SLC37) transporters (4). As these are very distinct MFS subfamilies MFSD1 seems to represent a new type of a hybrid SLC transporter carrying features from both families.

By combining targeted metabolomics and whole-cell patch-clamp experiments, we investigated the substrate spectrum and transport mode of MFSD1. We identified MFSD1 as a new type of highly selective lysine/arginine/histidine-containing dipeptide transporter, which functions as a uniporter to specifically export these dipeptides out of the lysosome.

## Results

### MFSD1 Mediates the Export of Lysosomal Dipeptides.

**Fig. 1.**
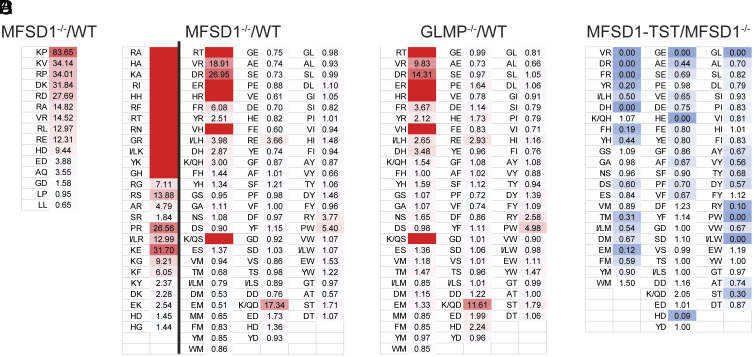
MFSD1-deficient lysosomes accumulate dipeptides. (*A*) Untargeted metabolomics analysis of lysosomes purified from WT and MFSD1^−/−^ murine colon carcinoma cell line MC-38. Accumulation of dipeptides (MFSD1^−/−^ vs. WT) is shown (n = 3 for both conditions). (*B*) Targeted dipeptide metabolomics analysis of lysosomes purified from WT and MFSD1^−/−^ 293LX cells. Accumulation of dipeptides (MFSD1^−/−^ vs. WT) is shown (n = 3 for both conditions). Fields with no number highlight dipeptides detected only in MFSD1^−/−^ lysosomes. Data are from two separate experiments, separated by a line, with additional dipeptides detected in the extra experiment in the left column. (*C*) Targeted dipeptide metabolomics analysis of lysosomes purified from WT and GLMLP^−/−^ 293LX cells. Accumulation of dipeptides (GLMP^−/−^ vs. WT) is shown (n = 3 for both conditions). Fields with no number highlight dipeptides detected only in GLMP^−/−^ lysosomes. (*D*) Targeted dipeptide metabolomics analysis of lysosomes purified from 293LX Tmem192-3xHA MFSD1^−/−^ ± MFSD1-TST cells. Rescue of dipeptide accumulation (MFSD1-TST vs. MFSD1^−/−^) is shown (n = 3 for both conditions).

Our previous studies demonstrating a crucial role for MFSD1 in regulating cell migration (7, 10), spurred us to try to identify its substrates. Since MFSD1 localizes to the lysosome (11) we examined the metabolites whose presence in this organelle changed upon MFSD1’s absence. For an initial untargeted metabolomics screen, the LysoIP protocol (12) was applied on murine colon carcinoma MC-38 WT and MFSD1^−/−^ cells, both expressing the tagged lysosomal protein Tmem192-3xHA for lysosome immunopurification. We observed a significant increase of dipeptides containing lysine and arginine in lysosomes of MFSD1^−/−^ cells when compared to control WT lysosomes (Fig. 1*A* and *SI Appendix*, Fig. S1 *A* and *B*), with a >80-fold increase for the dipeptide KP. For targeted metabolomics, we prepared WT and MFDS1^−/−^ human 293LX cells (*SI Appendix*, Fig. S1*C*) expressing the lysosomal bait protein Tmem192-3xHA (*SI Appendix*, Fig. S1*D*). We confirmed proper targeting by colocalization of Tmem192 with the lysosomal marker LAMP2 in both WT and MFSD1^−/−^ 293LX cells (*SI Appendix*, Fig. S1 *E* and *F*). The relative abundance of dipeptides in purified lysosomes from WT and MFSD1^−/−^ cells was compared to a generated library of 361 dipeptides. Strikingly, we observed a specific increase in the accumulation of dipeptides containing either arginine or lysine, and to a reduced degree also of histidine, while other dipeptides were not affected in MFSD1^−/−^ lysosomes (Fig. 1*B*). Interestingly, we also observed an accumulation of the single amino acids lysine and arginine in the purified MFSD1^−/−^ lysosomes from three different MFSD1^−/−^ single-cell clones, when compared to the corresponding WT single-cell clones (*SI Appendix*, Fig. S1*G*). From these initial targeted metabolomics experiments, we conclude that MFSD1 transports dipeptides containing at least one positively charged amino acid.

MFSD1 requires the presence of its accessory protein GLMP (13), and GLMP^−/−^ phenocopies MFSD1^−/−^ (7, 13). Nontargeted metabolomics of GLMP^−/−^ lysosomes showed the highest accumulation for positively charged dipeptides, with a 47-fold increase for the dipeptide RD (*SI Appendix*, Fig. S1*H*), thus, recapitulating the results obtained from the nontargeted analysis of MFSD1^−/−^ (*SI Appendix*, Fig. S1*B*). Additionally, we observed an increase in lipid metabolites in both, GLMP^−/−^ and MFSD1^−/−^, lysosomes (*SI Appendix*, Fig. S1 *B* and *H*), suggesting a compensatory process common to both knock-outs. We continued to perform a targeted metabolomics analysis on GLMP^−/−^ cells (*SI Appendix*, Fig. S1*C*). We found that the accumulation of arginine, lysine, and histidine-containing dipeptides in GLMP^−/−^ and MFDS1^−/−^ lysosomes was similar (Fig. 1*C*). Inducible re-expression of MFSD1 in 293LX Tmem192-3xHA MFSD1^−/−^ cells (*SI Appendix*, Fig. S1*I*) rescued dipeptide levels in these lysosomes to WT amount (Fig. 1*D*). These results suggest that MFSD1 likely functions as a dipeptide exporter.

### The Dipeptide KG-induced Currents Mediated by MFSD1.

**Fig. 2.**
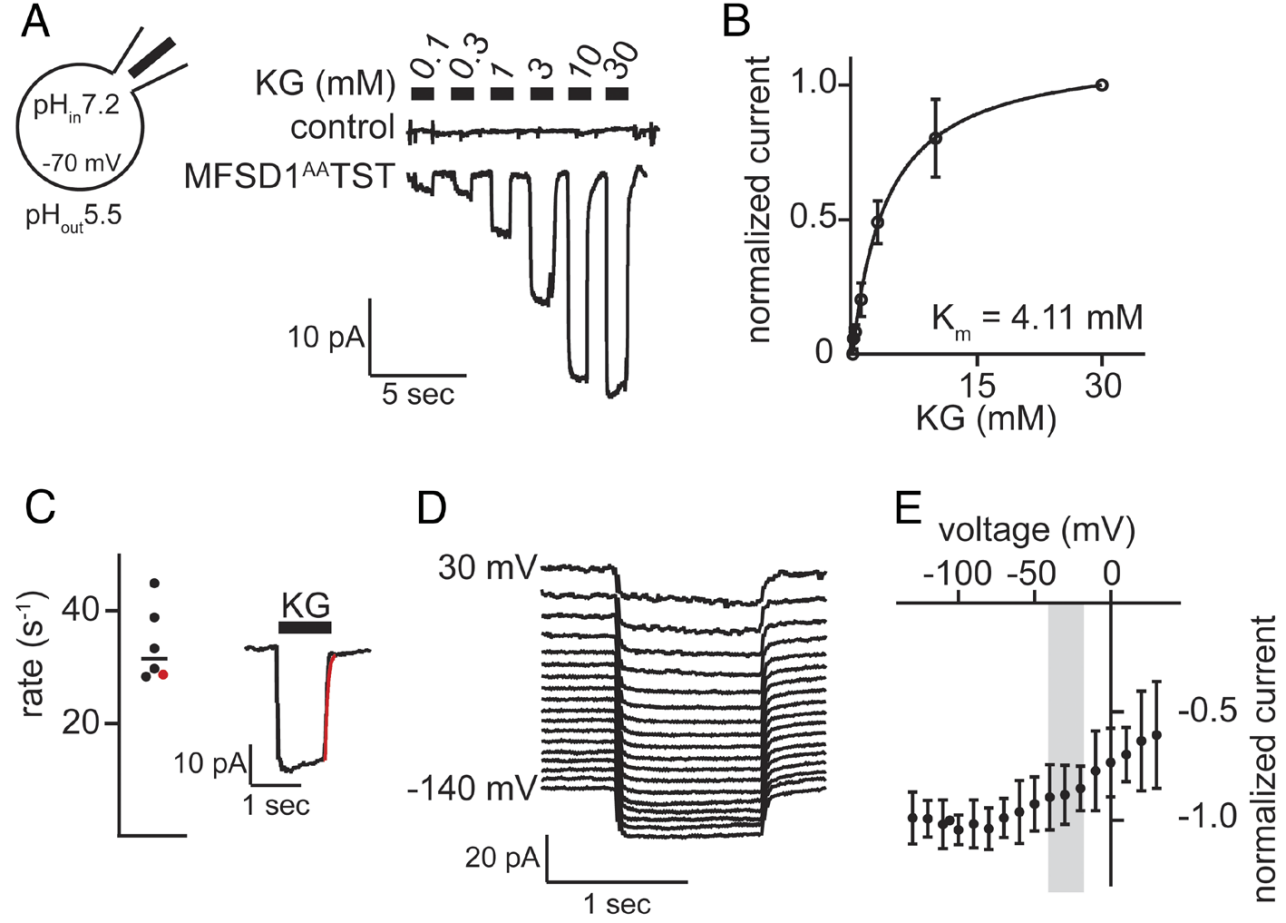
The dipeptide KG induces a MFSD1 dependent current. (*A*) Representative whole-cell patch-clamp recordings obtained from MFSD1^AA^TST expressing and control cells, respectively (n = 6). The cells were challenged with increasing KG concentrations. (*B*) Normalized current amplitudes induced by KG measured at −70 mV as a function of KG concentration (n = 6 cells). The solid line represents a fit of the Michaelis–Menten equation to the data points. (*C*) Current decay rates (n = 6). These were obtained by a fit of a monoexponential function to the current decay (see *Inset*: the fit is indicated in red. The red dot in the graph is the corresponding rate). (*D*) Representative currents induced by 3 mM KG measured in the voltage range between −140 mV to +30 mV. (*E*) Normalized current amplitudes as a function of the membrane potential (n = 5). The physiological range of the lysosomal membrane potential (17) is indicated by a gray box.

MFSD1 can be redirected to the plasma membrane by mutating its dileucine lysosomal targeting motif to alanine (13, 14) allowing patch clamp analysis. We confirmed the expected orientation of MFSD1^AA^ in the plasma membrane (i.e., lysosome luminal side exposed to the extracellular side, *SI Appendix*, Fig. S2*D*). Lysosomal pH conditions were achieved by setting extra- and intracellular pH to 5.5 and 7.2, respectively (*Inset* in Fig. 2*A*). The dipeptide KG was selected for further transport analysis on HEK293S GnT1^−/−^ cells expressing MFSD1^AA^TST. KG induced an inwardly directed current, which was absent in control cells (Fig. 2*A*). Its amplitude increased at higher concentrations of KG and started to level out at around 30 mM (Fig. 2*A*). A Michaelis–Menten fit to the normalized current amplitudes measured at −70 mV resulted in a K_m_ = 4.11 ± 0.41 mM (Fig. 2*B*). The rate of current deactivation upon removal of the substrate from the bath solution is a surrogate for the substrate turnover rate of a SLC (15, 16). A monoexponential fit to the current decay (see *Inset* in Fig. 2*C*, current deactivation indicated by the red line) yielded a decay rate of 34 ± 6 s^−1^ (Fig. 2*C*). In the voltage range between −140 mV to +30 mV (Fig. 2*D*) amplitudes were larger at negative potentials and leveled out above −80 mV (Fig. 2*E*). Under physiological lysosomal membrane voltages between −40 mV to −20 mV (17) MFSD1 operates at about 90 % of its maximal capacity.

### Positively Charged Dipeptides Are Substrates of MFSD1.

**Fig. 3.**
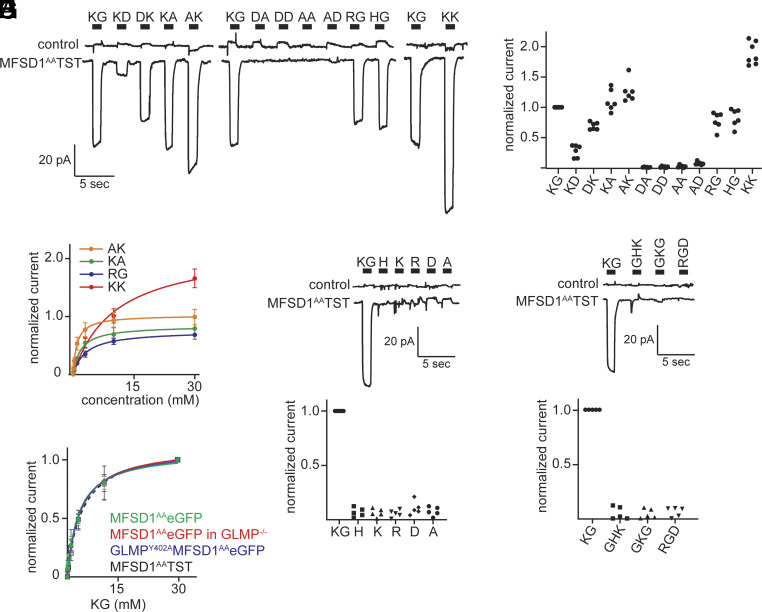
MFSD1 is a dipeptide transporter. (*A*) Representative whole-cell patch-clamp recordings obtained from MFSD1^AA^TST expressing and control cells, respectively. The cells were challenged with selected dipeptides applied at a concentration of 30 mM. (*B*) Summary of normalized current amplitudes induced by the tested dipeptides (normalized to 30 mM KG) (n ≥ 5). (*C*) Normalized amplitudes of currents evoked by selected dipeptides as a function of the concentration. The currents were normalized to the estimated current amplitude induced by a saturating concentration of KG. (*D*) Representative recordings obtained from MFSD1^AA^TST expressing and control cells, respectively (n = 5). The cells were challenged with KG (30 mM) and selected amino acids (20 mM). (*E*) Summary of normalized current amplitudes induced by the indicated amino acids (normalized to 30 mM KG) (n = 5). (*F*) Representative recordings obtained from MFSD1^AA^TST expressing and control cells, respectively (n = 5). The cells were challenged with KG (30 mM) and selected tripeptides (30 mM). (*G*) Summary of normalized current amplitudes evoked by the indicated tripeptides (normalized to 30 mM KG) (n = 5). (*H*) Normalized amplitudes of currents as a function of KG concentration measured at –70 mV from MFSD1^AA^eGFP in WT cells (green line, n = 7), MFSD1^AA^eGFP in GLMP^–/–^ cells (red line, n = 7), GLMP^Y402A^MFSD1^AA^eGFP in WT cells (purple line, n = 8), and MFSD1^AA^TST (dashed black line; the same as in Fig. 2*B*). The solid lines represent fits of the Michaelis–Menten equation to the data points.

A variety of positively and negatively charged as well as electroneutral (AA) dipeptides were tested as potential substrates using initial substrate concentrations of 30 mM. Lysine, arginine, and histidine-containing dipeptides induced inwardly directed currents (Fig. 3*A*). Dipeptides with a net negative charge (DA, DD, and AD), as well as the neutral dipeptide AA, failed to stimulate a current. Compared to the amplitudes induced by 30 mM KG only AK and KK created higher currents (Fig. 3 *A* and *B*), with the highest transport affinity for AK and KA with K_m_ = 0.97 ± 0.05 mM and 1.82 ± 0.11 mM (Table 1 and *SI Appendix*, Fig. S3 *A*–*F*). The different K_m_s partly explained the different current levels shown in Fig. 3*B*. At a concentration of 30 mM the current induced by KG, KA, AK, HG, RG, and KK, reaches about 88%, 94%, 97%, 92%, 91%, and 76% of the maximal amplitude, respectively.

**Table 1.** K_m_ ± SD values of tested dipeptides for MFSD1^AA^TST

dipeptide	K_m_ (mM)	±SD
KG	4.11	0.41
KG (pH_out_7.5)	3.90	0.20
RG	3.15	0.21
HG	2.23	0.58
KK	10.11	0.84
KK (pH_out_7.5)	3.36	0.16
DK	7.36	0.52
KA	1.82	0.11
AK	0.97	0.05

At a saturating dipeptide concentration, the current amplitude is determined by the substrate turnover rate and the charge of the substrate. To separate the distinct influences of these two factors, we plotted the concentration-dependent current amplitudes of RG, KA, AK, and KK (Fig. 3*C*), which are all positively charged (*SI Appendix*, Fig. S3*J*). When normalizing the respective current amplitude against the saturating V_max_ of KG, we obtained relative V_max_ values of RG, KA, AK, and KK of 0.76, 0.84, 1.03, and 2.2, respectively. The different dipeptides are transported at different rates but these differences are not large enough to obscure the influence of the substrate charge on the current amplitudes. Our results thus unequivocally show that MFSD1 is a dipeptide transporter, which can mediate the transit of dipeptides that contain at least one positively charged amino acid.

### Amino Acids and Tripeptides Are No Substrates of MFSD1.

We investigated whether MFSD1 could transport single amino acids or tripeptides. We applied three positively charged (H, K, and R), one negatively charged (D), and one neutral amino acid (A), at a concentration of 20 mM (Fig. 3 *D* and *E*). None of the tested amino acids was able to induce a current. This suggests that single amino acids are not substrates of MFSD1. The same holds true for tripeptides (GKG, RGD, and GHK) for which no currents were detected when applied at a concentration of 30 mM (Fig. 3 *F* and *G*). These data indicate a very high specificity of MFSD1 for dipeptides.

### GLMP Is Not Required for MFSD1 to Transport Dipeptides.

We sought to explore whether the transport function of MFSD1 differs in the presence or absence of GLMP. We first expressed MFSD1^AA^eGFP in WT and GLMP^−/−^ cells, respectively, and found that MFSD1^AA^eGFP was present at the plasma membrane of both cell lines (*SI Appendix,* Fig. S2*A*). Notably, the current amplitudes evoked by the dipeptide KG and the K_m_ values in WT and GLMP^−/−^ cells were indistinguishable (Fig. 3*H*, Table 2, and *SI Appendix*, Fig. S3 *G* and *H*). We also expressed the fusion protein GLMP^Y402A^MFSD1^AA^eGFP in WT cells and confirmed cell surface localization (*SI Appendix*, Fig. S2*B*) and proper orientation (i.e., lysosome luminal side exposed to the extracellular side) (*SI Appendix*, Fig. S2*E*). As seen, the fusion protein GLMP^Y402A^MFSD1^AA^eGFP and MFSD1^AA^eGFP had essentially the same K_m_ value for the dipeptide KG (Fig. 3*H*, Table 2, and *SI Appendix*, Fig. S3*I*). These data, therefore, suggest that MFSD1 requires GLMP to resist lysosomal degradation (13, 18) but not for dipeptide transport.

**Table 2.** K_m_ ± SD values for KG of different MFSD1 constructs

	KG	
	K_m_ (mM)	±SD
MFSD1^AA^eGFP in WT cells	3.38	0.26
MFSD1^AA^eGFP in GLMP^−/−^ cells	3.59	0.16
GLMP^Y402A^MFSD1^AA^eGFP in WT cells	3.08	0.39

### The Role of Protons in the Transport Cycle of MFSD1.

**Fig. 4.**
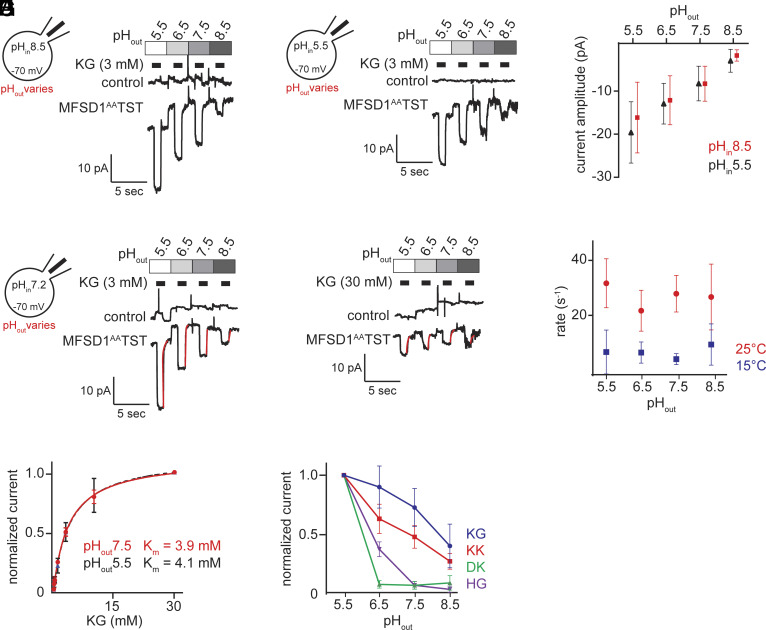
MFSD1 transport efficiency of particular dipeptides depends on lysosomal pH due to changes in substrate protonation. (*A*) Whole-cell patch-clamp recordings of currents induced by 3 mM KG at pH_in_8.5 and varying pH_out_ values which correspond to the pH on the lysosomal side (n = 5 cells). (*B*) The same as in *A* but with pH_in_5.5. (*C*) Current amplitude (pA) as a function of pH_out_ for pH_in_5.5 (in black) and pH_in_8.5 (in red), respectively. (*D*) Representative current traces recorded at 25 °C (pH_in_7.2 and varying pH_out_) (the red lines are fits of a monoexponential function to the current decay). (*E*) The same as in *D* but recorded at 15 °C. Because of the lower temperature, we had to apply 30 mM KG to obtain currents with sufficient amplitude. (*F*) Decay rates obtained from experiments such as in *D* and *E* as a function of pH_out_ at 25 °C (red, n = 5) and 15 °C (blue, n = 8), respectively. (*G*) Normalized currents induced by KG as a function of KG concentration at pH_out_5.5 and pH_out_7.5 (n = 5 cells), respectively. The solid red line is a fit of the Michaelis–Menten equation to the data points. The dashed black line (pH_out_5.5) is the same as in Fig. 2*B*. (*H*) Normalized currents induced by 3 mM KG, KK, DK, and HG, respectively (pH_in_7.2 and varying pH_out_ values; n = 5 for KG, n = 6 for KK and HG, n = 8 for DK).

Dipeptide transporters such as SLC15A1-A5 or their bacterial homologue DtpB are known to be proton-coupled (19, 20). The proton gradient across the lysosomal membrane would suggest that MFSD1 might as well be proton-driven, although recently lysosomal uniporters were identified (21). We varied extra- and intracellular proton concentrations to investigate the pH dependence of MFSD1-mediated currents induced by 3 mM KG at a membrane potential of −70 mV. We found that a successive increase of the pH from 5.5 to 8.5 on the cis-side (i.e., the side from which the substrate is applied) led to a reduction of the current amplitude. Conversely, changing the pH on the trans-side (i.e., the intracellular side) from 5.5 to 8.5 had no appreciable effect on the current amplitude (Fig. 4 *A*–*C*). However, if a proton was an obligatory cosubstrate of MFSD1, raising the proton concentration intracellularly ought to have reduced the current amplitude. This is because the proton can rebind to the transporter after it is released and thereby hamper the progress of the transport cycle. Rebinding, will more frequently occur if the proton concentration is high. The absence of current inhibition upon a raise in proton concentration by a factor of 1,000 (from pH 8.5. to 5.5), thus, refutes the idea that protons are obligatory cosubstrates of MFSD1 (Fig. 4*C*). Hence, we conclude that MFSD1 is a uniporter.

We then explored alternate hypotheses for the influence of pH on the current amplitude. We reasoned that increases in pH could be decreasing the turnover of substrates by deprotonating one of the transporter’s acidic amino acids. We examined the current deactivation rate as a surrogate for the rate of substrate turnover and determined whether it was inhibited at a higher pH. We extracted current deactivation rates from experiments conducted both at 25 °C (Fig. 4*D*) and 15 °C (Fig. 4*E*), to slow down the transport kinetics of MFSD1 and thus better resolve rate differences. While the deactivation rate was reduced at 15 °C, it was unaffected by changes in pH_out_ (Fig. 4*F*). These data indicate that increases in pH do not alter the transporter’s capacity to turnover substrate.

We then hypothesized that the deleterious effects of higher pH on current amplitude could be due to i) a loss in substrate affinity or ii) a change of the charge carried by the substrate. We first determined the K_m_ of KG at pH_out_7.5. At this pH_out_ the K_m_ of KG was essentially the same as that at pH_out_5.5 (Fig. 4*G* and *SI Appendix*, Fig. S4*A* and Table 1). Then we turned to potential alterations in the charge carried by the dipeptides. The pK_A_ value of a dipeptide depends on its amino acid composition. Accordingly, if the loss of the current amplitude at higher pH_out_ values is caused by a change in the protonation state of the transported substrate, the proton concentration dependence of this effect ought to vary between the different tested dipeptides. Indeed, we observed a varying decline in the currents evoked by the dipeptides upon increasing the pH_out_ (Fig. 4*H* and *SI Appendix*, Fig. S4 *B*–*E*), with a rank order by pKa value of DK < HG < KK < KG. The observation that the pH dependence of the current amplitude is a property of the chosen substrate supports the idea that the decline in current amplitude at higher pH_out_ values is due to the gradual deprotonation of the dipeptide. For KK we confirmed that the reduction in current amplitude at higher pH values was not caused by a loss of its affinity for MFSD1 (*SI Appendix*, Fig. S4 *F* and *G* and Table 1). This could not be tested for DK and HG because these dipeptides failed to produce a current at higher pH_out_ values.

In summary, the data presented here show that MFSD1 functions as a uniporter and that its transport rate is not affected by the ambient pH.

## Discussion

In this study, we investigated the functional properties of the lysosomal MFSD1 transporter. We used two complementary experimental approaches to identify and verify MFSD1’s substrates: targeted metabolomics and whole-cell patch-clamp electrophysiological recordings. Our findings revealed that MFSD1 acts as a uniporter, driven by the substrate gradient, exporting lysine/arginine/histidine-containing dipeptides out of the lysosome under physiological conditions. The step of substrate release is not proton-dependent, and the substrate transport is equally efficient against or down a pH gradient.

Our measurements revealed that MFSD1 reaches its full capacity already at modest negative voltages. This is notable because most transporters residing on the plasma membrane approach this point at more negative membrane potentials. For instance, in the case of the dopamine transporter, the transport rate at −80 mV is approximately 5 times higher than at 0 mV (22). In contrast, for MFSD1 this rate increases only by a factor of about 1.3 over the same voltage range and KG was transported at about 75% of its maximal rate at 0 mV.

From the dipeptides investigated by electrophysiology, a combination of charged residue (K/R/H) and neutral residue (A/G) showed K_m_ values ranging from 0.9 to 4 mM, while neutral dipeptides (DK) or double-charged dipeptides (KK) showed significantly lower transport affinities of 7 to 10 mM. Neither amino acids nor tripeptides were substrates of MFSD1. MFSD1 when driven by the dipeptide gradient is per se thermodynamically uncoupled from the H^+^ gradient. Acidic conditions in our in vitro experiments ensured substrate protonation, but pH did not affect the substrate turnover rate. These functional properties of MFSD1 are clearly different from the mammalian dipeptide transporters of the SLC15 family.

The five members in the SLC15 family form the group of mammalian proton-coupled oligopeptide transporters (POT). SLC15A1 and SLC15A2 have been extensively studied due to their long-recognized clinical importance (23), including the uptake of drugs into the intestine, kidney, and brain, making them extremely important for effective pharmacological interventions (24, 25). SLC15A1 and SLC15A2 share a vast substrate spectrum, which is typical for peptide transporters (19, 26–28). Two additional members, SLC15A3 and A4, have been investigated (29, 30); SLC15A3 and SLC15A4 can transport dipeptides, especially the dipeptide carnosine, yet they have a higher transport rate for histidine (31–33). Both proteins display the dileucine motif required for lysosomal targeting (14) and are found in lysosomes (29, 34). SLC15A3 and A4 are preferentially expressed in leukocytes and their involvement in immune-related functions has recently been highlighted (34–37).

MFSD1 exhibits low overall sequence similarities to mammalian and bacterial POT transporters (4). MFS transporters are characterized by the arrangement of their 12 transmembrane helices into four triplets. Triplet 1 to 2 forms the 6-TM N-terminal bundle and triplet 3 to 4 the C-terminal bundle. The AlphaFold-model of MFSD1 (AF-Q9H3U5-F1-model_v4.pdb) suggests structural similarities to the bacterial homologues of the POT family, such as PepT_St_ from *Streptococcus thermophilus* (pdb entry codes 5OXL, 5OXM, 5OXN, 5OXO) (*SI Appendix*, Fig. S5*A*), YePEPT from *Yersinia enterocolitica* (4WSV.pdb), the mammalian-like bacterial POT family transporter from *Xanthomonas campestris*, PepT_Xc_ (6EL3.pdb), GkPOT from *Geobacillus kaustophilus* (4IKV.pdb), and PepT_So_ from S*hewanella oneidensis*. Different from these bacterial POTs, MFSD1 does not comprise the TM helices HA and HB linking the N- and C-terminal bundles that form the substrate binding site. Instead, in the AlphaFold prediction of MFSD1, N- and C-terminal bundles are linked via an extended cytoplasmic loop (*SI Appendix*, Fig. S5*B*), which is only slightly reminiscent of the helical folded loop in mammalian POTs (*SI Appendix*, Fig. S5*C*).

POT transporters generally display a high substrate promiscuity (8). The peptide binding site is well conserved throughout evolution (38), and both, the flexibility of the peptide binding site and of the substrate, in addition with versatile water coordination, contribute to the observed promiscuity (20, 26, 27, 38–41). Specifically, the binding site in POT transporters comprises several hydrophobic residues forming pockets accommodating the side chains of peptides, which are less pronounced in MFSD1. The formation and dissolution of these pockets are linked to the different binding modes of peptides (40). Docking of the dipeptides RG, KG, or HG to the AlphaFold prediction of MFSD1 suggests a binding site at a comparable position to bacterial POTs, *e.g.*, to the Phe-Ala or Asp-Glu binding in PepT_St_ (pdb entries 5OXM and 5OXN) (*SI Appendix*, Fig. S5 *D* and *E*). MFSD1 comprises at least four tyrosine residues close to the putative binding pocket. Positively charged residues contributing to substrate coordination of negatively charged dipeptides, such as Arg26 and Ly126 in PepT_St_ to coordinate Asp-Glu (*SI Appendix*, Fig. S5*E*) are replaced in MFSD1 by Glu151 (*SI Appendix*, Fig. S5 *F* and *G*), which would support an increased affinity for positively charged dipeptides.

For the bacterial POTs potential proton binding sites were suggested to be located in TM1 and TM7, the first helix of triplet 1 and triplet 3, respectively. MFSD1 lacks the ExxERFxYY motif in TM1, which is present in all POT family members (42), and essential for proton-driven uptake of dipeptides (43, 44). This is in agreement with the observed functional differences of MFSD1 and POT transporters.

Lysosomes serve as degradative organelles, digesting macromolecules including proteins. A failure in the degradation of macromolecules or export of digested products can lead to impaired lysosomal functioning, eventually culminating in lysosomal storage disorders (45). The export of dipeptides from lysosomes was reported (46–51), however, the identity of the membrane proteins responsible for the observed transport remained unknown (52). In this work, we describe for the first time the relative amounts of dipeptides in lysosomes and the identity of one of the long-posited dipeptide transporters. A striking increase in the levels of dipeptides containing arginine, lysine, and to a minor extent also histidine was detected in MFSD1^−/−^ and GLMP^−/−^ lysosomes when compared to wild-type controls. MFSD1^AA^eGFP expressed in GLMP^−/−^ cells displayed the same transport characteristics as MFSD1^AA^eGFP expressed in WT cells indicating that GLMP does not affect the transport function. We therefore conclude that the sole function of the accessory protein GLMP is to ensure the protein stability of its nonglycosylated interaction partner MFSD1 within the lysosomal membrane (7, 13, 18).

To date, it is still an open question how these specific dipeptides exported by MFSD1 contribute to the phenotypes observed in the MFSD1^−/−^ mouse (13), in MFSD1^−/−^ tumor cells (7), and during lymphocyte development and liver homeostasis (53). Dipeptides have been shown to influence the activity of enzymes (54–58), to affect cell migration (59, 60), to play a role as antioxidants (61), to exhibit antidepressant-like activity (62), and to be involved in the eradication of cytotoxic lymphocytes (63, 64). We speculate that MFSD1 might be implicated in at least some of these actions, and that its function is not limited to providing amino acids for reuse in protein synthesis.

## Materials and Methods

### Cell Lines.

MC-38 and HEK293 Lenti-X cells (Takara, #632180, Kusatsu, Japan) were cultured in Dulbecco’s Modified Eagle’s Medium (DMEM) (Thermo Fisher Scientific, #31966-021) supplemented with 10% Fetal Calf Serum (FCS) (Sigma, #9665) and incubated at 37 °C with 5% CO_2_. HEK293S GnT1^−/−^ cells (ATCC, #CRL-3022, Manassas, USA) were cultivated in DMEM-Ham’s F12 (Thermo Fisher Scientific, #10565-018) supplemented with 10% FCS and incubated at 37 °C with 5% CO_2_. The expression of ectopic MFSD1 variants in respective cells was induced with Doxycycline for 18 to 24 h.

### Construct Cloning.

The constructs used in this study are listed in *SI Appendix,* Table S1. All restriction enzymes and T4 ligase used for cloning were from New England Biolabs (NEB, Ipswitch, USA) and the Gateway BP and LR Clonase II were from Thermo Fisher Scientific. All Gateway cloning constructs were cloned via the donor plasmid pDONR221 (Addgene, #12536017, Watertown, USA) into the Doxycycline inducible expression plasmid pINDUCER20 (65). Constructs were heat-shock transformed into One Shot Stbl3 *E. coli* bacteria (Thermo Fisher Scientific, #C737303). Proper sequence of all constructs has been confirmed by sequencing (Microsynth, Balgach, Switzerland) (*SI Appendix*, Table S1).

Constructs generated for mammalian expression were separately packed into lentiviral particles using pdelta8.9 (Addgene, #2221) and pCMV-VSV-G (Addgene, #8454) cotransfected using Lipofectamine 3000 (Thermo Fisher Scientific) into Lenti-X 293T cells (TaKaRa, #632180). Crude lentiviral supernatant was used for infection of target mammalian cells. Cells were selected for stable integration of the constructs via respective antibiotic selection.

### Generation of MFSD1^−/−^ and GLMP^−/−^ Cells.

The generation of MC-38 WT and MFSD1^−/−^ is described elsewhere (7). HEK293 Lenti-X (Takara, #632180) were seeded on a 24-well plate and at ~80% confluency cells were transfected with Lipofectamine CRISPRMAX Cas9 Transfection reagent, as suggested by the manufacturer (Qiagen, #CMAX00001, Hilden, Germany) and shown in *SI Appendix,* Table S2. All RNA oligos were from Integrated DNA Technologies (IDT).

One day after transfection cells were expanded on a T25 Tissue culture flask (TPP, #90026). Additional 3 d later, cells were single-cell cloned into 96-well plates. Two to three weeks later single-cell clones were determined with an Olympus CKX41 microscope with a Olympus Plan C N 4×/0.10 objective (Olympus, Shinjuku, Japan) and expanded. MFSD1^−/−^ and GLMP^−/−^ was verified by qPCR as follows: RNA was isolated from single-cell clones using the RNeasy Mini Kit (Qiagen, #74104) according to manufacturers’ instructions. cDNA synthesis and qPCR was performed in a single tube using the Luna Universal One-Step RT-qPCR Kit (NEB, #E3005) with an input of 0.1 µg to 1.0 µg of total isolated RNA per reaction on a LightCycler 480 machine (Roche, Basel, Switzerland) using primers listed in *SI Appendix,* Table S3.

### Lysosomal Purification using Tmem192-3xHA Tagged Lysosomes (Lyso-IP).

MC-38 and HEK293 Lenti-X WT, MFSD1^−/−^, MFSD1^−/−^::MFSD1-TST (rescue cell line), and GLMP^−/−^ cells were stably transfected to express the HA-tagged lysosomal marker Tmem192, suitable for immunopurification of intact lysosomes (12). Specifically, cells were expanded onto three p100 Tissue Culture Dishes (VWR, #734-2321), washed with cold PBS and scraped into 2 mL Potassium Phosphate Buffered Saline (KPBS) (136 mM KCl, 10 mM KH_2_PO_4_, pH 7.25) and centrifuge for 2 min, at 1,000 × g, 4 °C. The cell pellet was resuspended in 1 mL KPBS and cells were homogenized with a glass dounce homogenizer, applying 20 strokes. The cell lysate was cleared by centrifugation for 2 min, at 1,000 × g, 4 °C. The cell lysate containing the lysosomes was added to 100 µL pre-equilibrated Peirce Anti-HA Magnetic beads (Thermo Fisher Scientific, #88836) and incubated rotating at 4 °C for 3 min. The beads were washed three times with 1 mL KPBS, and the lysosomal metabolites were extracted using 50 µL of Extraction buffer (methanol/acetonitrile/ water = 40/40/20) for 10 min at room temperature. The beads were removed and the extract was snap-frozen and stored at −80 °C until further analysis.

### Nontargeted Mass Spectrometry.

Data-dependent LC–MS/MS (liquid chromatography-tandem mass spectrometry) was performed with an Ultimate 3000 High-Performance Liquid Chromatography (HPLC) system, coupled to a Q Exactive Focus mass spectrometer (both Thermo Fisher Scientific) via electrospray ionization. A flow rate of 100 μL/min was used and 1 μL of each sample was injected onto the respective column and guard column. For reversed phase chromatography, an ACQUITY UPLC HSS T3 (Waters; 150 mm × 2.1 mm; 1.8 μm) was used. Employing a 20 min linear gradient of 99% A (0.1% formic acid in water) to 60% B (acetonitrile with 0.1% formic acid). In HILIC (hydrophilic interaction chromatography), a SeQuant ZiC-pHILIC, (Merck; 5 µm, 100 × 2.1 mm) was used with a 20 min gradient from 90% A (acetonitrile) to 80% B (20 mM ammonia bicarbonate in water). Mass spectra were acquired with a resolution of 70,000 in polarity switching mode with full MS acquisition from m/z 70 to m/z 1,045. MS/MS data were collected in data-dependent acquisition mode in a pooled quality control sample, containing aliquots of all samples and using a normalized collision energy of 25. Data analysis and statistical evaluation was performed with Compound Discoverer (Thermo Fisher Scientific).

### Targeted Mass Spectrometry.

Detection and quantification of amino acids and dipeptides was done by LC–MS/MS, using a Vanquish HPLC system coupled to a TSQ Altis mass spectrometer (both Thermo Fisher Scientific), employing the Selected Reaction Monitoring (SRM) mode and positive polarity. In brief, dried samples were resolved in 0.1% formic acid in water and 1 µL was injected onto a Kinetex (Phenomenex) C18 column (100 Å, 150 × 2.1 mm), employing a 9-min-long linear gradient from 100% A (1% acetonitrile, 0.1% formic acid in water) to 90% B (0.1% formic acid in acetonitrile) at a flow rate of 100 µL/min. Retention times, SRM transitions, and optimal collisional energies were determined by authentic standards and degenerate dipeptide libraries. All data interpretation was performed using Xcalibur (Thermo Fisher Scientific).

### Western Blotting.

Cell lysates or purified lysosomes were separated on 4 to 15% Sodium Dodecyl Sulfate - PolyAcrylamide Gel Electrophoresis (SDS-PAGE) gradient gels (Bio-Rad, Hercules, USA) and transferred on Protran 0.45 nitrocellulose membranes (GE Healthcare, Chicago, USA). The nitrocellulose membrane was blocked with 1× Pierce Clear Milk Blocking Buffer (Thermo Fisher Scientific, #37587) for 1 h at room temperature. Primary antibodies were incubated in blocking buffer overnight at 4 °C, while secondary antibodies were incubated in blocking buffer for 1 h at room temperature. Following washing with Tris Buffered Saline-Tween 20 (TBS-T), membranes were incubated with SuperSignal West Femto Maximum Sensitivity Substrate (Thermo Fisher Scientific, #34096) and the chemiluminescent signal was detected with the ChemiDoc MP Gel Imaging System (Bio-Rad). Antibodies used are listed in *SI Appendix,* Table S4.

### Immunofluorescence.

Cells were grown on Nunc Lab-Tek Chamber Slides (Thermo Fisher Scientific, #154534) and recombinant gene expression was induced by 100 ng/µL doxycycline for 24 h. Cells were fixed with 4% formaldehyde in PBS (Thermo Fisher Scientific, #28906) for 10 min at room temperature (RT) followed by blocking/permeabilization with 1% BSA/0.1% Triton X-100 in PBS for 1 h at RT. Cells were stained with primary antibodies in blocking/permeabilization buffer for 2 h at RT, followed by secondary antibody staining for 1 h at RT. Cells were counterstained with DAPI for 10 min at RT and mounted in ProLong Diamond Antifade Mountant (Thermo Fisher Scientific, #P36970). Between incubation steps cells were washed with PBS for 3 × 5 min. Immunofluorescent pictures were taken on a Zeiss LSM880 inv. Fast Airyscan confocal microscope with a Plan-Apochromat ×40/NA 1.3 OIL objective. Colocalization (Pearson’s R value with the Coloc 2 tool) was determined by Image J. Antibodies used are listed in *SI Appendix,* Table S4.

### Live Cell Fluorescence Imaging.

Cells were grown on poly-D-lysine coated glass-bottom dishes and expression of MFSD1^AA^eGFP or GLMP^Y402A^MFSD1^AA^eGFP was induced for 24 h with 5 µg/mL Doxycycline. Prior confocal imaging, cells were rinsed in Live cell imaging solution (Gibco, #A59688DJ), and stained with 0.4% Trypan Blue solution (Sigma, T8154) for 3 min at room temperature. Cells were washed three times with Live cell imaging solution and confocal pictures were taken on a Nikon Eclipse Ti (Nikon, Minato, Japan) confocal microscope with a Plan-Apochromat VC ×60/NA 1.4 Oil DIC N2 objective. Images were processed by Image J.

### Flow Cytometry.

Cells were washed off the cell culture plate and washed once in cold Fluorescence Activated Cell Sorting (FACS) buffer (1% BSA, 10 mM Ethylenediaminetetraacetic acid in PBS). Cells were incubated for 10 min on ice with Fc block (BioLegend, #422301) and stained with anti-myc 4A6 (Sigma-Aldrich, #05-724) or anti-GFP 5G4 (E. Ogris lab, MPL Vienna) for 30 min. Cells were washed once with FACS buffer and stained with secondary anti-mouse AF633 antibody (Thermo Fisher Scientific, #A21050) for additional 30 min. After the final wash, cells were resuspended in 250 µL FACS buffer and analyzed on a Beckton Dickinson CytoFLEX S (Beckton Dickinson, Franklin Lakes, USA) flow cytometer. Data were processed by FlowJo software (Tree Star Inc., Ashland, USA).

### Whole-Cell Patch-Clamp Electrophysiology.

HEK293S GnT1^−/−^ cells expressing hsMFSD1^AA^TST, HEK293 Lenti-X wild-type or GLMP^−/−^ cells expressing hsMFSD1^AA^eGFP, and HEK293 Lenti-X wild-type cells expressing the fusion protein GLMP^Y402A^MFSD1^AA^eGFP upon Doxycycline induction for 18 to 24 h, or control, were grown on poly-D-lysine coated cell culture dishes. During experiments, cells were maintained in a buffer consisting of 150 mM NaCl, 3 mM KCl, 2.5 mM CaCl_2_, 2 mM MgCl_2_, 20 mM glucose, and 10 mM 4-(2-hydroxyethyl)-1-piperazineethanesulfonic acid (HEPES), with the pH adjusted to 7.4 using NaOH.

The patch pipette solution was composed of 150 mM NMDG (N-methyl-D-glucamine), 1 mM CaCl_2_, 0.7 mM MgCl_2_, 10 mM HEPES, and 10 mM ethylene glycol-bis(β-aminoethyl ether)-N,N,N′,N′-tetraacetic acid. The pH was titrated to 5.5, 7.2, or 8.5 with MsOH (methanesulfonic acid) or NMDG.

For dipeptide application, the buffer comprised 150 mM NMDG, 2.5 mM CaCl_2_, 2 mM MgCl_2_, 20 mM glucose, and, depending on the desired pH, 10 mM MES (2-(N-morpholino)ethanesulfonic acid) for pH 5.5 and for pH 6.5, 10 mM HEPES for pH 7.5, and 10 mM tris(hydroxymethyl)aminomethane for pH 8.5. Peptides and amino acids were purchased at Bachem Holding AG (Bubendorf, Switzerland), Carl Roth GmbH + Co (Karlsruhe, Germany), or Sigma-Aldrich (St. Louis, MO, USA), respectively (*SI Appendix,* Table S5). All buffers were prepared freshly before each experiment. All chemicals utilized were of analytical grade and were employed without further purification, sourced from Sigma-Aldrich (St. Louis, MO, USA) unless otherwise specified.

Dipeptides were applied via a perfusion system (Octaflow II, ALA Scientific Instruments, Inc.), which allowed for rapid and complete solution exchange around the cells within a 20-ms timeframe.

Experiments were conducted at 15 °C or 25 °C, as indicated, with precise temperature control provided by a temperature control unit (cell microcontrol inline preheater, Green Leaf Scientific), which was linked to a temperature control system (PTC-20, npi electronic GmbH, Tamm, Germany). Both, the patch-clamp pipette, and the perfusion system were manipulated using the PatchStar micromanipulator (Scientifica Ltd., Uckfield, East Sussex, United Kingdom).

Patch-clamp recordings were conducted in the whole-cell configuration using an Axon 200B amplifier equipped with an Axon 1550 digitizer. Recordings were sampled at 100 µs intervals, and current amplitudes and associated kinetics were analyzed using Clampfit software (Molecular Devices, LLC, San José, CA, USA). Passive holding currents were subtracted, and the traces were filtered using an 80 Hz digital 8-pole Bessel low-pass filter.

### Statistical Analysis.

All analyses were performed using Graph Pad Prism version 9 (GraphPad Software, Boston, MA, USA). Data shown are as mean ± SD.

## Supplementary Material

Appendix 01 (PDF)

## Data Availability

All study data are included in the article and/or *SI Appendix*.
