# Supplementary material for: Orphan lysosomal solute carrier MFSD1 facilitates highly selective dipeptide transport
Source: Proc Natl Acad Sci U S A. 2024 Mar 20;121(13):e2319686121. doi: 10.1073/pnas.2319686121 (PMC10990142; doi:10.1073/pnas.2319686121)
Supplement: Supplementary file 1 — Appendix 01 (PDF) [file pnas.2319686121.sapp.pdf]

PNAS

Supporting Information for
Orphan lysosomal solute carrier MFSD1 facilitates highly selective dipeptide transport

Danila Boytsov[1], Gregor Madej[2], Georg Horn[2], Nadine Blaha[3], Thomas Köcher[3], Harald H. Sitte[1,4,5], Daria Siekhaus[6,7,8], Christine Ziegler[2,8], Walter Sandtner[1,8], Marko Roblek[1,6]*

Corresponding author: Marko Roblek
Email: marko.roblek@meduniwien.ac.at

This PDF file includes:

Figures S1 to S5
Tables S1 to S5

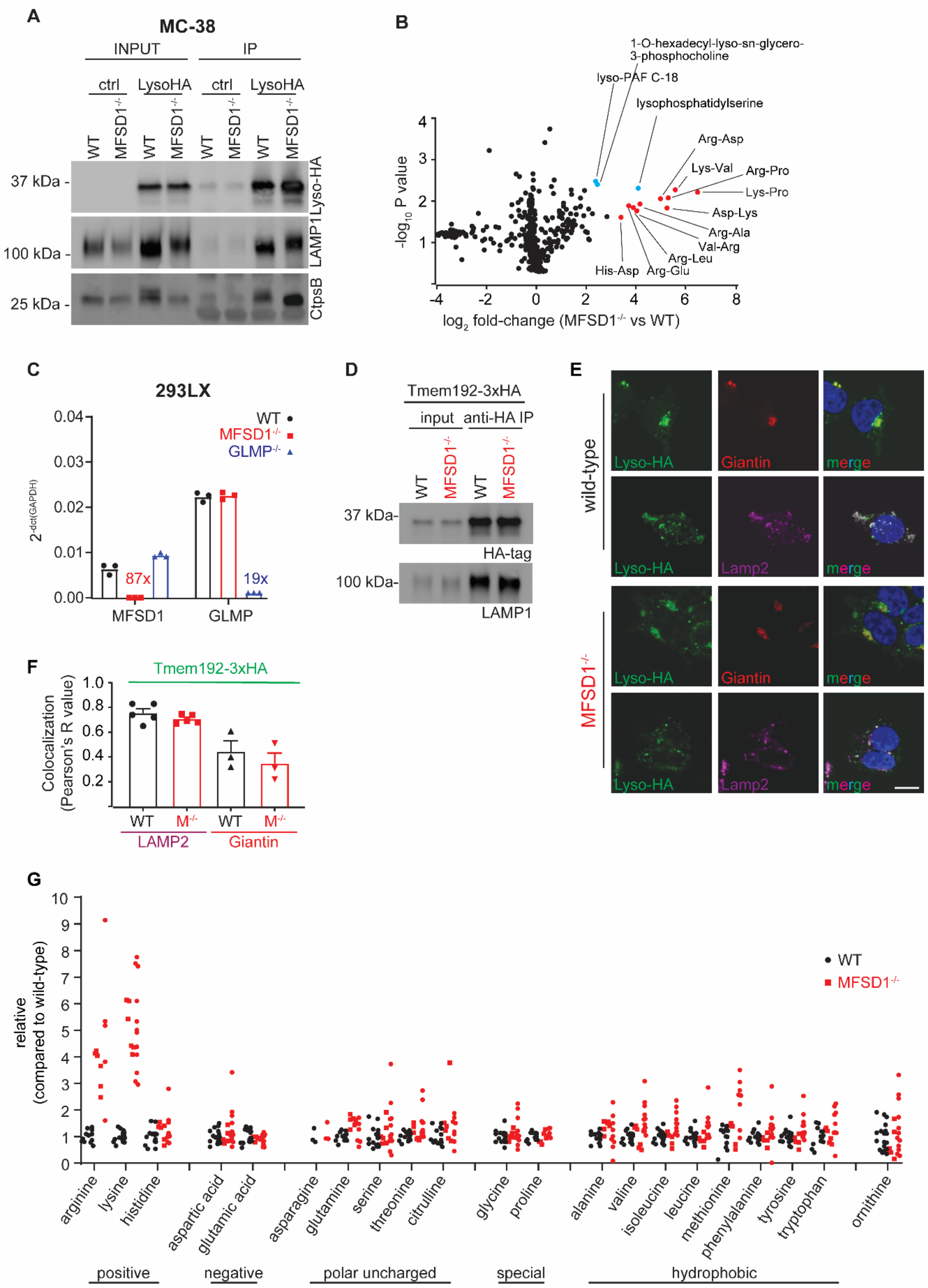

A
MC-38
INPUT
IP
ctrl
LysoHA
WT
MFSD1-/-
37 kDa -
100 kDa -
25 kDa -
Lyso-HA
LAMP1
CtpsB
B
1-O-hexadecyl-lyso-sn-glycero-3-phosphocholine
lyso-PAF C-18
lysophosphatidylserine
Arg-Asp
Lys-Val
Arg-Pro
Lys-Pro
Asp-Lys
Arg-Ala
Val-Arg
Arg-Leu
His-Asp
Arg-Glu
-log10 P value
log2 fold-change (MFSD1-/- vs WT)
C
293LX
WT
MFSD1-/-
GLMP-/-
87x
19x
MFSD1
GLMP
2-dct(GAPDH)
D
Tmem192-3xHA
input
anti-HA IP
37 kDa-
100 kDa-
HA-tag
LAMP1
E
wild-type
MFSD1-/-
Lyso-HA
Giantin
Lamp2
merge
F
Tmem192-3xHA
Colocalization (Pearson's R value)
WT
M-/-
LAMP2
Giantin
G
relative (compared to wild-type)
WT
MFSD1-/-
arginine
lysine
histidine
aspartic acid
glutamic acid
asparagine
glutamine
serine
threonine
citrulline
glycine
proline
alanine
valine
isoleucine
leucine
methionine
phenylalanine
tyrosine
tryptophan
ornithine
positive
negative
polar uncharged
special
hydrophobic

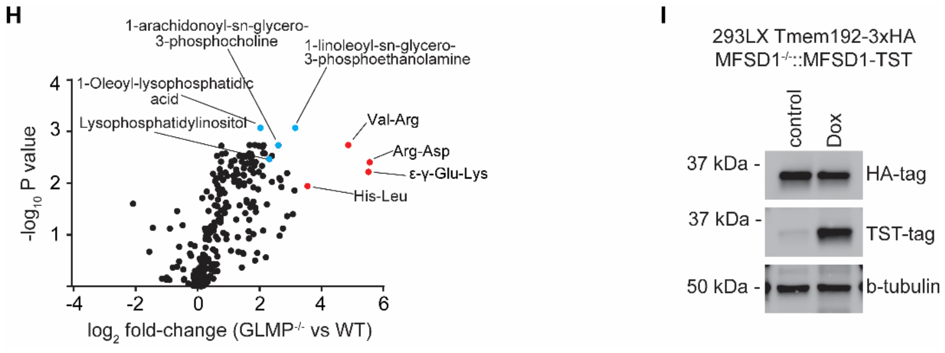

Figure S1. MFSD1-deficient lysosomes accumulate dipeptides. A) Western blot of lysosomal marker Tmem192-3xHA immunoprecipitation from MC-38 Tmem192-3xHA WT and MFSD1$^{-/-}$ cells. Co-immunoprecipitation of lysosomal marker LAMP1 and lysosomal cathepsin B is shown. **B)** Untargeted metabolomics analysis of lysosomes purified from WT and MFSD1$^{-/-}$ MC-38 cells. Log_2-fold change of metabolites (MFSD1$^{-/-}$ vs WT) with respective p-values is shown (n=3 for both condition). **C)** Confirmation of MFSD1$^{-/-}$ and GLMP$^{-/-}$ knockout by q-PCR of each in 293LX WT, MFSD1$^{-/-}$, and GLMP$^{-/-}$ cells, respectively. The mean fold reduction in respective mRNA levels is indicated (n=3). **D)** Western blot of lysosomal marker Tmem192-3xHA immunoprecipitation from 293LX Tmem192-3xHA WT and MFSD1$^{-/-}$ cells. Co-immunoprecipitation of lysosomal marker LAMP1 is shown. **E)** Co-immunofluorescence of Tmem192-3xHA with Giantin (Golgi-marker) and LAMP2 (lysosomal marker) in 293LX WT and MFSD1$^{-/-}$ cells. Scale bar = 10 µm. **F)** Co-localization analysis of Giantin and LAMP2 with Tmem192-3xHA in 293LX WT and MFSD1$^{-/-}$ cells (n=5 for WT and MFSD1$^{-/-}$ for LAMP2; n=3 for WT and MFSD1$^{-/-}$ for Giantin). **G)** Targeted amino acid metabolomics analysis of lysosomes purified from three independent WT and MFSD1$^{-/-}$ 293LX Tmem192-3xHA single cell clones (n≥3 for each WT and MFSD1$^{-/-}$ clone). **H)** Untargeted metabolomics analysis of lysosomes purified from WT and GLMP$^{-/-}$ 293LX cells. Log_2-fold change of metabolites (GLMP$^{-/-}$ vs WT) with respective p-values is shown (n=3 for both condition). **I)** Western blot of 293LX Tmem192-3xHA MFSD1$^{-/-}$ cells re-expressing MFSD1-TST upon 1 µg/ml Doxycycline treatment for 24 hours.

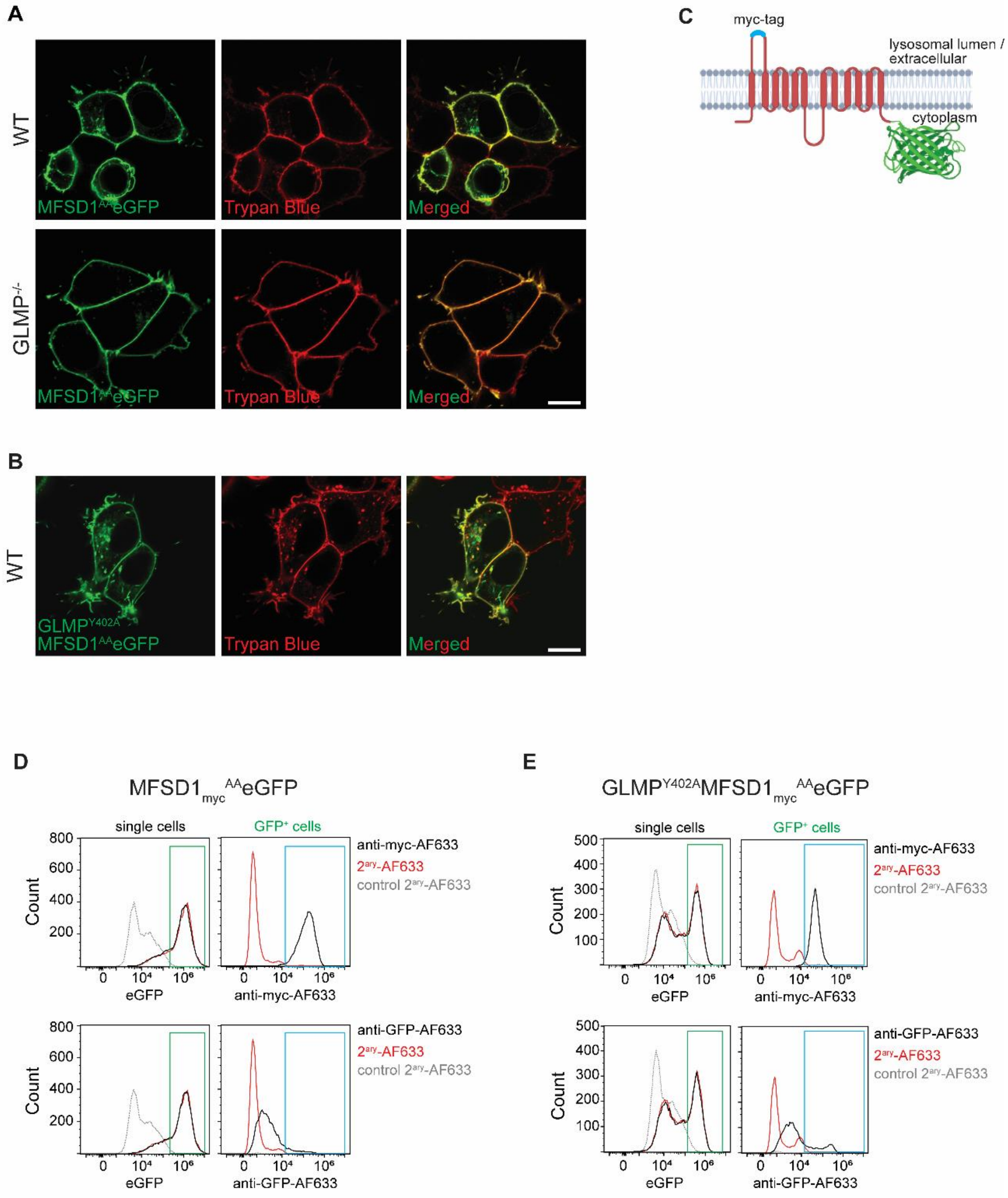
A
WT
GLMP-/-
MFSD1AAeGFP
Trypan Blue
Merged
B
WT
GLMPY402A
MFSD1AAeGFP
Trypan Blue
Merged
C
myc-tag
lysosomal lumen /
extracellular
cytoplasm
D
MFSD1mycAAeGFP
single cells
GFP+ cells
Count
eGFP
anti-myc-AF633
anti-GFP-AF633
2ary-AF633
control 2ary-AF633
E
GLMPY402AMFSD1mycAAeGFP
single cells
GFP+ cells
Count
eGFP
anti-myc-AF633
anti-GFP-AF633
2ary-AF633
control 2ary-AF633

Figure S2. MFSD1^{AA} variants localize to the cell membrane, with the N- and C- termini facing the cytoplasm. A) Live cell confocal images of MFSD1^{AA}eGFP in WT and GLMP$^{-/-}$ cells. Cell membrane was stained by Trypan blue (n=6). **B)** Live cell confocal images of GLMP^{Y402A}MFSD1^{AA}eGFP in WT cells. Cell membrane was stained by Trypan blue (n=6). Scale bar in B and C = 10 µm. **C)** Cartoon of MFSD1$_{myc}^{AA}$eGFP topology. MFSD1 (red), myc-tag (blue), eGFP (green) are indicated (done by BioRender). **D)** Flow cytometry analysis of MFSD1$_{myc}^{AA}$eGFP topology. GFP^{+} cells (green gate) were analyzed for anti-myc-AF633 (top panel) or anti-GFP-AF633 (bottom panel) signal. Myc-AF633^{+} or GFP-AF633^{+} gates are indicated with the pale blue box (n=3). **E)** Flow cytometry analysis of GLMP^{Y402A}MFSD1$_{myc}^{AA}$eGFP topology. GFP^{+} cells (green gate) were analyzed for anti-myc-AF633 (top panel) or anti-GFP-AF633 (bottom panel) signal. Myc-AF633^{+} or GFP-AF633^{+} gates are indicated with the pale blue box (n=3).

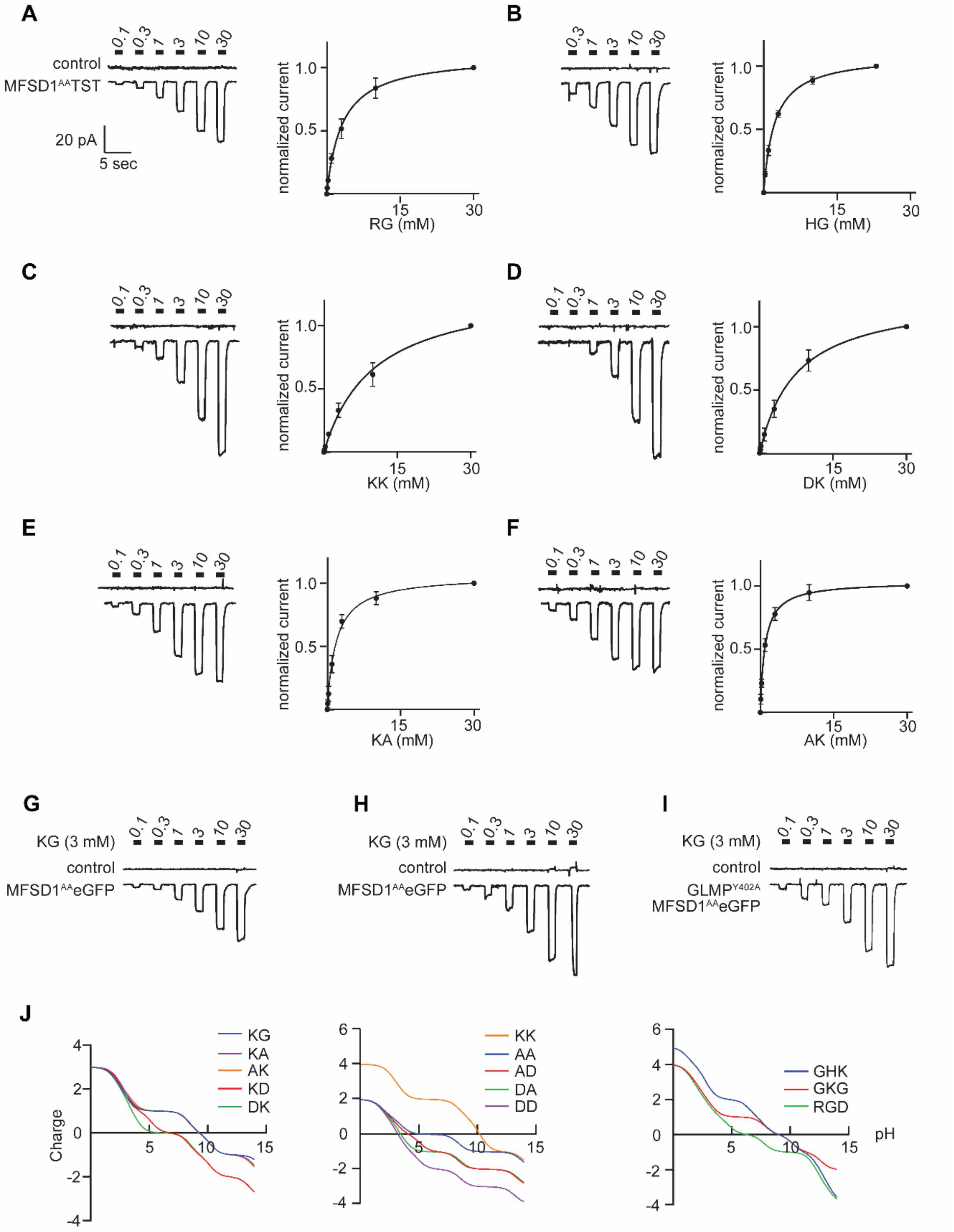
A
0.1 0.3 1 3 10 30
control
MFSD1^AA TST
20 pA
5 sec
normalized current
1.0
0.5
15
30
RG (mM)
B
0.3 1 3 10 30
normalized current
HG (mM)
C
KK (mM)
D
DK (mM)
E
KA (mM)
F
AK (mM)
G
KG (3 mM)
control
MFSD1^AA eGFP
H
I
GLMP^Y402A
MFSD1^AA eGFP
J
Charge
KG
KA
AK
KD
DK
KK
AA
AD
DA
DD
GHK
GKG
RGD
pH

Figure S3. MFSD1 is a dipeptide transporter. A-F) Whole-cell patch-clamp concentration-dependent current recordings of indicated dipeptides in control and $MFSD1^{AA}$TST expressing cells, respectively (n≥6). Labeling in A) applies to A-F. **G)** Whole-cell patch-clamp concentration-dependent current recordings of KG in control and $MFSD1^{AA}$eGFP expressing wild-type cells, respectively (n=7). **H)** Whole-cell patch-clamp concentration-dependent current recordings of KG in control and $MFSD1^{AA}$eGFP expressing $GLMP^{-/-}$ cells, respectively (n=7). **I)** Whole-cell patch-clamp concentration-dependent current recordings of KG in control and $GLMP^{Y402A}MFSD1^{AA}$eGFP expressing wild-type cells, respectively (n=8). Scale in A) applies to A-I. **J)** pH dependent net charge of dipeptides and tripeptides used in this study. Graphs were obtained from https://chemicalize.com.

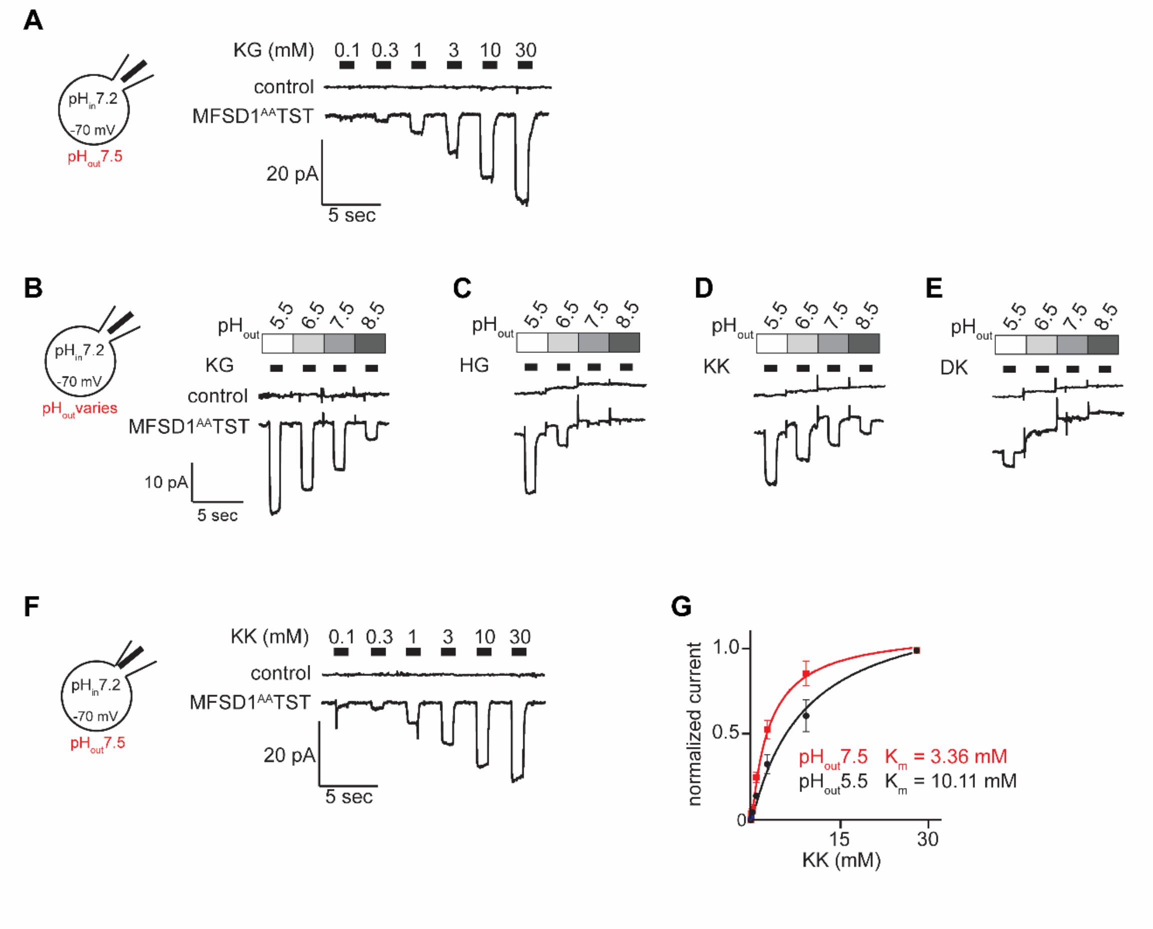

Figure S4. K_m of KG for MFSD1 is independent of pH_{out}. A) Whole-cell patch-clamp concentration-dependent current recording at pH_{in}7.2 and pH_{out}7.5 of KG in control and MFSD1^{AA}TST expressing cells, respectively. Current recording from one representative measurement is shown (n=6). **B-E)** Representative whole-cell patch-clamp current traces for **B)** 3 mM KG (n=5), **C)** 3 mM HG (n=6), **D)** 3 mM KK (n=6), and **E)** 3 mM DK (n=8) at pH_{in}7.2 and varying pH_{out} values. Labeling and scale in B) applies to B-E. **F)** Whole-cell patch-clamp concentration-dependent current recording at pH_{in}7.2 and pH_{out}7.5 of KK in control and MFSD1^{AA}TST expressing cells, respectively. Current recording from one representative measurement is shown (n=10). **G)** Normalized currents induced by KK as function of KK concentration at pH_{out}5.5 (n=7; the same as in *SI Appendix*, Fig. S3C) and pH_{out}7.5 (n=10 cells), respectively. The solid lines are a fit of the Michaelis Menten equation to the data points.

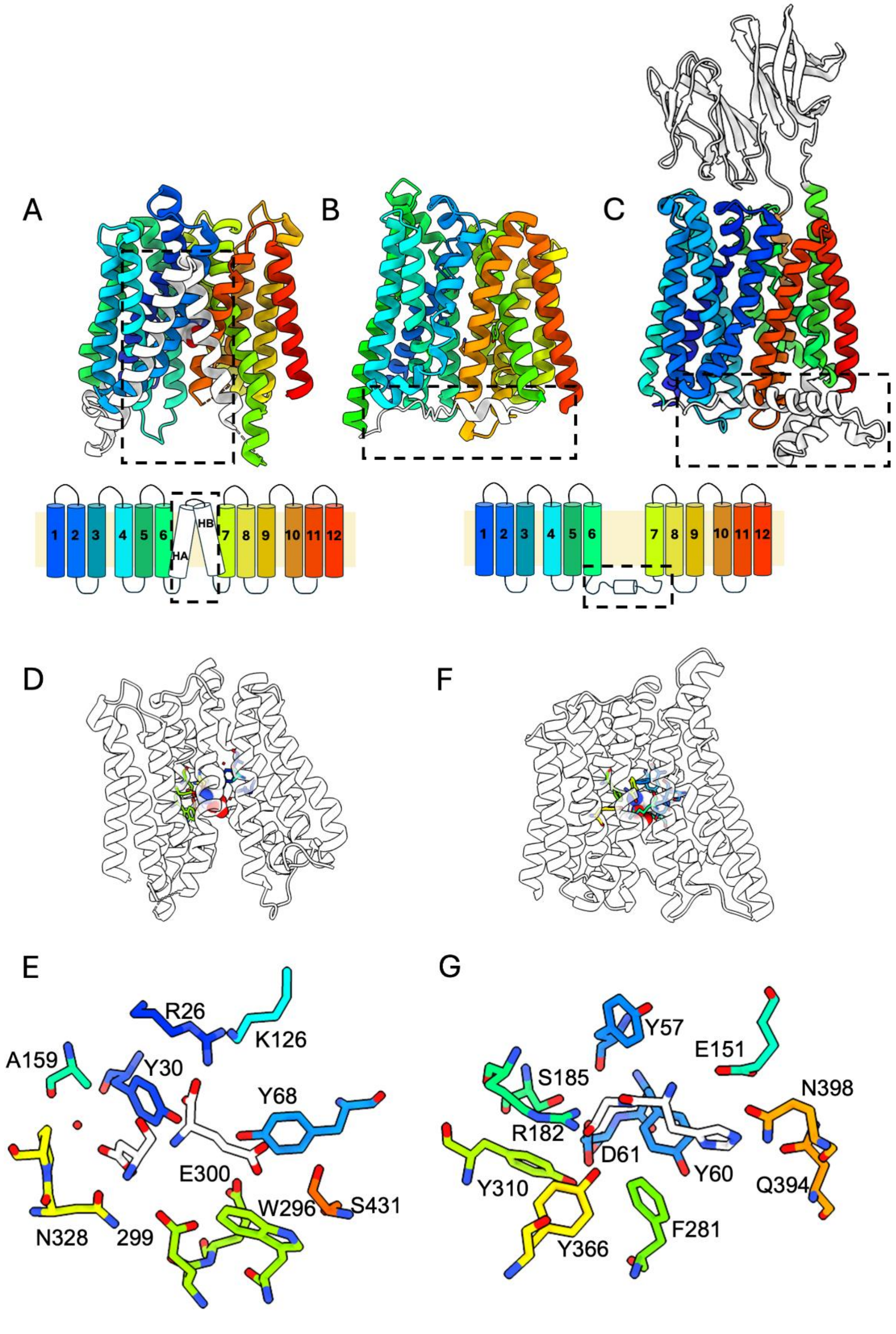
A
B
C
1
2
3
4
5
6
HB
HA
7
8
9
10
11
12
D
F
E
R26
K126
A159
Y30
Y68
E300
W296
S431
N328
299
G
Y57
E151
S185
N398
R182
D61
Y60
Q394
Y310
F281
Y366

Figure S5. Structural comparison between homologues of the POT family and MFSD1. A) X-ray structure of $PepT_{St}$ from *Streptococcus thermophilus* in an inward-facing state in (5OXO.pdb), a representative bacterial homologue of the POT family. View of the HA and HB helices (colored in white, dashed box) connecting the N-terminal bundle (TM1-TM6) and the C-terminal bundle (TM7-TM12). HA and HB are a characteristic feature of bacterial POTs; also shown in the topology in similar color coding. **B)** AlphaFold model of MFSD1 (Q9H3U5, AF-Q9H3U5-F1-model_v4.pdb) in an inward-facing state with N ($\Delta 38$)- and C ($\Delta 23$) -terminal domains omitted from the figure for clarity. N- and C-terminal bundles are predicted to be connected by a loop (Leu 243-Leu 264, colored in white, dashed box) comprising a helical segment; shown in the topology below. **C)** Cryo-EM structure of hsPepT2, a mammalian homologue of the POT family (7PMY.pdb) in an inward-facing state. An extracellular immunoglobulin-like domain (ECD) is placed between TM9 and TM10. N- and C- terminal bundles are connected via a long cytoplasmic linker, comprising a helical hairpin common for all SLC15 transporters. **D)** X-ray structure of $PepT_{St}$ in an inward-facing state in complex with Asp-Glu (5OXM.pdb) viewed 180° turned in comparison to the structure displayed in A). **E)** Zoom into the binding site of $PepT_{St}$ coordinating Asp-Glu (5OXM.pdb). Residues are color-coded according to the topology model. **F)** Docking of His-Gly to the AlphaFold model of MFSD1 (Q9H3U5, AF-Q9H3U5-F1-model_v4.pdb) in an inward-facing state. **G)** Zoom into the predicted binding site of the AlphaFold model of MFSD1. Residues are color-coded according to the topology model.

Table S1. Plasmids used in this study. Primer sequences, template plasmids, and backbone plasmids used to clone the final plasmids used in this study is shown. Sequences of final constructs, shown below, have been confirmed by sequencing.

FINAL PLASMID	PRIMERS	ENZYMES	TEMPLATE	BACKBONE
pInducer20-hsMFSD1-Twin-Strep-Tag	5’-GGG GAC AAG TTT GTA CAA AAA AGC AGG CTT AAT GGA GGA GGA GGA C-3’	BP and LR Clonase II	pDonR221_ MFSD1 (Addgene, #131916)	pInducer20 (2)
	5’-GGG GAC CAC TTT GTA CAA GAA AGC TGG GTA TTA ctt ctc aaa ttg tgg atg aga cca ggc aga acc tcc cga tcc acc tcc gga acc tcc acc ttt ttc gaa ttg tgg atg act cca agc act acc CTC GGT GTG AGA GAA C-3'			
pLJC5-Tmem192-3xHA			Addgene, #102930 (1)	
pInducer20-hsMFSD1-AA-Twin-Strep-Tag	5’-gggg aca agt ttg tac aaa aaa gca ggc tta ATG GAG GAG GAG GAC GAG GAG GCA CGG GCC gca gca GCA GGA GGA CCC GAC-3’	BP and LR Clonase II	pDonR221_ MFSD1 (Addgene, #131916)	pInducer20 (2)
	5’-GGG GAC CAC TTT GTA CAA GAA AGC TGG GTA TTA ctt ctc aaa ttg tgg atg aga cca ggc aga acc tcc cga tcc acc tcc gga acc tcc acc ttt ttc gaa ttg tgg atg act cca agc act acc CTC GGT GTG AGA GAA C-3'			
phsMFSD1-eGFP-N1	5'-gat ctc gag ATG GAG GAG GAG GAC-3'	XhoI	pDonR221_ MFSD1 (Addgene, #131916)	peGFP-N1 (Addgene, #6085-1)
	5'-c gac cgg taa CTC GGT GTG AGA GAA C-3'	AgeI		
pInducer20-hsMFSD1-AA-eGFP	5'-gggg aca agt ttg tac aaa aaa gca ggc tta ATG GAG GAG GAG GAC GAG GAG GCA CGG GCC gca gca GCA GGA GGA CCC GAC-3'	BP and LR Clonase II	phsMFSD1-eGFP-N1	pInducer20 (2)
	5'-GGGG AC CAC TTT GTA CAA GAA AGC TGG GTA TTA CTT GTA CAG CTC-3'			
pInducer20-hsGLMP^{Y402A} Gly-linker hsMFSD1-AA-eGFP	5'- gggg aca agt ttg tac aaa aaa gca ggc tta ATG CGC GGC TCT GTG GAG-3'		TFORF3513 (Addgene, #112770)	
	5'-ggc aga acc tcc cga tcc acc tcc gga acc tcc acc ATT TAT GGA CTG Ggc CTC TGA GTA CTT CTT G-3'			
	5'-ggt gga tcg gga ggt tct gcc ATG GAG GAG GAG GAC-3'		pInducer20-hsMFSD1-AA-eGFP	
	5'-GGGG AC CAC TTT GTA CAA GAA AGC TGG GTA TTA CTT GTA CAG CTC-3'			
	5'- gggg aca agt ttg tac aaa aaa gca ggc tta ATG CGC GGC TCT GTG GAG-3'	BP and LR Clonase II	PCR products from former two reactions	pInducer20 (2)
	5'-GGGG AC CAC TTT GTA CAA GAA AGC TGG GTA TTA CTT GTA CAG CTC-3'			

pInducer20-hsMFSD1^{2xmyc}-AA-eGFP	5'-gggg aca agt ttg tac aaa aaa gca ggc tta ATG GAG GAG GAG GAC GAG GAG GCA CGG GCC gca gca GCA GGA GGA CCC GAC-3'		pInducer20-hsMFSD1-AA-eGFP	
	5'-GAGGACCTGGAGCAGAAACTCATCTCAGAAGAAGACCTGggaggaggtgctGTGAATACCACAAAGTTTATG-3'			
	5'-GTTTCTGCTCCAGGTCCTCTTCGCTAATGAGTTTCTGTTCagagcctccaccTTGCATATCCCGCTTCACC-3'		pInducer20-hsMFSD1-AA-eGFP	
	5'-GGGG AC CAC TTT GTA CAA GAA AGC TGG GTA TTA CTT GTA CAG CTC-3'			
	5'-gggg aca agt ttg tac aaa aaa gca ggc tta ATG GAG GAG GAG GAC GAG GAG GCA CGG GCC gca gca GCA GGA GGA CCC GAC-3'	BP and LR Clonase II	PCR products from former two reactions	pInducer20 (2)
	5'-GGGG AC CAC TTT GTA CAA GAA AGC TGG GTA TTA CTT GTA CAG CTC-3'			
pInducer20-hsGLMP^{Y402A} Gly-linker hsMFSD1^{2xmyc}-AA-eGFP	5'-gggg aca agt ttg tac aaa aaa gca ggc tta ATG GAG GAG GAG GAC GAG GAG GCA CGG GCC gca gca GCA GGA GGA CCC GAC-3'		pInducer20-hsGLMP^{Y402A} Gly-linker hsMFSD1-AA-eGFP	
	5'-GAGGACCTGGAGCAGAAACTCATCTCAGAAGAAGACCTGggaggaggtgctGTGAATACCACAAAGTTTATG-3'			
	5'-GTTTCTGCTCCAGGTCCTCTTCGCTAATGAGTTTCTGTTCagagcctccaccTTGCATATCCCGCTTCACC-3'		pInducer20-hsGLMP^{Y402A} Gly-linker hsMFSD1-AA-eGFP	
	5'-GGGG AC CAC TTT GTA CAA GAA AGC TGG GTA TTA CTT GTA CAG CTC-3'			
	5'- gggg aca agt ttg tac aaa aaa gca ggc tta ATG CGC GGC TCT GTG GAG-3'	BP and LR Clonase II	PCR products from former two reactions	pInducer20 (2)
	5'-GGGG AC CAC TTT GTA CAA GAA AGC TGG GTA TTA CTT GTA CAG CTC-3'			

hsMFSD1-TST

ATGGAGGAGGAGGACGAGGAGGCACGGGCCCTGCTGGCAGGAGGACCCGACGAGGCCGATAGAGGCGCCCC
CGCCGCCCCTGGCGCCCTGCCTGCCCTGTGCGATCCATCTCGGCTGGCCCACAGACTGCTGGTGCTGCTGC
TGATGTGCTTCCTGGGCTTTGGCAGCTACTTCTGTTATGACAACCCTGCCGCCCTGCAGACCCAGGTGAAG
CGGGATATGCAAGTGAATACCACAAAGTTTATGCTGCTGTACGCCTGGTATTCCTGGCCAAACGTGGTGCT
GTGCTTCTTCGGCGGCTTCCTGATCGACAGGGTGTTTGGCATCCGCTGGGGCACAATCATCTTCTCTTGCT
TCGTGTGCATCGGACAGGTGGTGTTCGCACTGGGAGGCATCTTCAACGCCTTTTGGCTGATGGAGTTTGGC
CGGTTCGTGTTTGGAATCGGAGGAGAGAGCCTGGCAGTGGCACAGAACACCTACGCCGTGTCCTGGTTCAA
GGGCAAGGAGCTGAATCTGGTGTTTGGCCTGCAGCTGTCCATGGCCAGAATCGGCTCTACAGTGAACATGA
ATCTGATGGGCTGGCTGTATAGCAAGATCGAGGCCCTGCTGGGATCCGCCGGACACACCACACTGGGAATC
ACCCTGATGATCGGCGGCATCACATGCATCCTGAGCCTGATCTGTGCCCTGGCCCTGGCCTACCTGGATCA
GAGGGCCGAGCGCATCCTGCACAAGGAGCAGGGCAAGACCGGCGAAGTGATCAAGCTGACAGACGTGAAGG
ATTTCTCCCTGCCACTGTGGCTGATCTTTATCATCTGCGTGTGCTACTATGTGGCCGTGTTCCCCTTTATC
GGCCTGGGCAAGGTGTTCTTTACCGAGAAGTTCGGCTTTAGCTCCCAGGCCGCCAGCGCCATCAATTCCGT
GGTGTATGTGATCTCTGCCCCAATGAGCCCCGTGTTCGGCCTGCTGGTGGACAAGACAGGCAAGAACATCA
TCTGGGTGCTGTGCGCAGTGGCAGCCACCCTGGTGAGCCACATGATGCTGGCCTTTACAATGTGGAATCCT
TGGATCGCCATGTGCCTGCTGGGCCTGTCTTACAGCCTGCTGGCATGTGCACTGTGGCCAATGGTGGCCTT
CGTGGTCCCTGAGCACCAGCTGGGAACCGCCTATGGCTTTATGCAGTCTATCCAGAACCTGGGCCTGGCCA
TCATCTCTATCATCGCCGGCATGATCCTGGATAGCAGGGGCTACCTGTTCCTGGAGGTGTTCTTTATCGCC
TGCGTGAGCCTGTCTCTGCTGTCCGTGGTGCTGCTGTACCTGGTGAATCGCGCCCAGGGCGGCAACCTGAA
TTATTCCGCCCGGCAGAGAGAGGAGATCAAGTTCTCTCACACCGAGggtagtgcttggagtcatccacaat
tcgaaaaaggtggaggttccggaggtggatcgggaggttctgcctggtctcatccacaatttgagaagtaa

hsMFSD1^{AA}TST

ATGGAGGAGGAGGACGAGGAGGCACGGGCCgcagcaGCAGGAGGACCCGACGAGGCCGATAGAGGCGCCCC
CGCCGCCCCTGGCGCCCTGCCTGCCCTGTGCGATCCATCTCGGCTGGCCCACAGACTGCTGGTGCTGCTGC
TGATGTGCTTCCTGGGCTTTGGCAGCTACTTCTGTTATGACAACCCTGCCGCCCTGCAGACCCAGGTGAAG
CGGGATATGCAAGTGAATACCACAAAGTTTATGCTGCTGTACGCCTGGTATTCCTGGCCAAACGTGGTGCT
GTGCTTCTTCGGCGGCTTCCTGATCGACAGGGTGTTTGGCATCCGCTGGGGCACAATCATCTTCTCTTGCT
TCGTGTGCATCGGACAGGTGGTGTTCGCACTGGGAGGCATCTTCAACGCCTTTTGGCTGATGGAGTTTGGC
CGGTTCGTGTTTGGAATCGGAGGAGAGAGCCTGGCAGTGGCACAGAACACCTACGCCGTGTCCTGGTTCAA
GGGCAAGGAGCTGAATCTGGTGTTTGGCCTGCAGCTGTCCATGGCCAGAATCGGCTCTACAGTGAACATGA
ATCTGATGGGCTGGCTGTATAGCAAGATCGAGGCCCTGCTGGGATCCGCCGGACACACCACACTGGGAATC
ACCCTGATGATCGGCGGCATCACATGCATCCTGAGCCTGATCTGTGCCCTGGCCCTGGCCTACCTGGATCA
GAGGGCCGAGCGCATCCTGCACAAGGAGCAGGGCAAGACCGGCGAAGTGATCAAGCTGACAGACGTGAAGG
ATTTCTCCCTGCCACTGTGGCTGATCTTTATCATCTGCGTGTGCTACTATGTGGCCGTGTTCCCCTTTATC
GGCCTGGGCAAGGTGTTCTTTACCGAGAAGTTCGGCTTTAGCTCCCAGGCCGCCAGCGCCATCAATTCCGT
GGTGTATGTGATCTCTGCCCCAATGAGCCCCGTGTTCGGCCTGCTGGTGGACAAGACAGGCAAGAACATCA
TCTGGGTGCTGTGCGCAGTGGCAGCCACCCTGGTGAGCCACATGATGCTGGCCTTTACAATGTGGAATCCT
TGGATCGCCATGTGCCTGCTGGGCCTGTCTTACAGCCTGCTGGCATGTGCACTGTGGCCAATGGTGGCCTT
CGTGGTCCCTGAGCACCAGCTGGGAACCGCCTATGGCTTTATGCAGTCTATCCAGAACCTGGGCCTGGCCA
TCATCTCTATCATCGCCGGCATGATCCTGGATAGCAGGGGCTACCTGTTCCTGGAGGTGTTCTTTATCGCC
TGCGTGAGCCTGTCTCTGCTGTCCGTGGTGCTGCTGTACCTGGTGAATCGCGCCCAGGGCGGCAACCTGAA
TTATTCCGCCCGGCAGAGAGAGGAGATCAAGTTCTCTCACACCGAGggtagtgcttggagtcatccacaat
tcgaaaaaggtggaggttccggaggtggatcgggaggttctgcctggtctcatccacaatttgagaagtaa

hsMFSD1^AA^eGFP

ATGGAGGAGGAGGACGAGGAGGCACGGGCCgcagcaGCAGGAGGACCCGACGAGGCCGATAGAGGCGCCCC
CGCCGCCCCTGGCGCCCTGCCTGCCCTGTGCGATCCATCTCGGCTGGCCCACAGACTGCTGGTGCTGCTGC
TGATGTGCTTCCTGGGCTTTGGCAGCTACTTCTGTTATGACAACCCTGCCGCCCTGCAGACCCAGGTGAAG
CGGGATATGCAAGTGAATACCACAAAGTTTATGCTGCTGTACGCCTGGTATTCCTGGCCAAACGTGGTGCT
GTGCTTCTTCGGCGGCTTCCTGATCGACAGGGTGTTTGGCATCCGCTGGGGCACAATCATCTTCTCTTGCT
TCGTGTGCATCGGACAGGTGGTGTTCGCACTGGGAGGCATCTTCAACGCCTTTTGGCTGATGGAGTTTGGC
CGGTTCGTGTTTGGAATCGGAGGAGAGAGCCTGGCAGTGGCACAGAACACCTACGCCGTGTCCTGGTTCAA
GGGCAAGGAGCTGAATCTGGTGTTTGGCCTGCAGCTGTCCATGGCCAGAATCGGCTCTACAGTGAACATGA
ATCTGATGGGCTGGCTGTATAGCAAGATCGAGGCCCTGCTGGGATCCGCCGGACACACCACACTGGGAATC
ACCCTGATGATCGGCGGCATCACATGCATCCTGAGCCTGATCTGTGCCCTGGCCCTGGCCTACCTGGATCA
GAGGGCCGAGCGCATCCTGCACAAGGAGCAGGGCAAGACCGGCGAAGTGATCAAGCTGACAGACGTGAAGG
ATTTCTCCCTGCCACTGTGGCTGATCTTTATCATCTGCGTGTGCTACTATGTGGCCGTGTTCCCCTTTATC
GGCCTGGGCAAGGTGTTCTTTACCGAGAAGTTCGGCTTTAGCTCCCAGGCCGCCAGCGCCATCAATTCCGT
GGTGTATGTGATCTCTGCCCCAATGAGCCCCGTGTTCGGCCTGCTGGTGGACAAGACAGGCAAGAACATCA
TCTGGGTGCTGTGCGCAGTGGCAGCCACCCTGGTGAGCCACATGATGCTGGCCTTTACAATGTGGAATCCT
TGGATCGCCATGTGCCTGCTGGGCCTGTCTTACAGCCTGCTGGCATGTGCACTGTGGCCAATGGTGGCCTT
CGTGGTCCCTGAGCACCAGCTGGGAACCGCCTATGGCTTTATGCAGTCTATCCAGAACCTGGGCCTGGCCA
TCATCTCTATCATCGCCGGCATGATCCTGGATAGCAGGGGCTACCTGTTCCTGGAGGTGTTCTTTATCGCC
TGCGTGAGCCTGTCTCTGCTGTCCGTGGTGCTGCTGTACCTGGTGAATCGCGCCCAGGGCGGCAACCTGAA
TTATTCCGCCCGGCAGAGAGAGGAGATCAAGTTCTCTCACACCGAGttaccggtcgccaccATGGTGAGCA
AGGGCGAGGAGCTGTTCACCGGGGTGGTGCCCATCCTGGTCGAGCTGGACGGCGACGTAAACGGCCACAAG
TTCAGCGTGTCCGGCGAGGGCGAGGGCGATGCCACCTACGGCAAGCTGACCCTGAAGTTCATCTGCACCAC
CGGCAAGCTGCCCGTGCCCTGGCCCACCCTCGTGACCACCCTGACCTACGGCGTGCAGTGCTTCAGCCGCT
ACCCCGACCACATGAAGCAGCACGACTTCTTCAAGTCCGCCATGCCCGAAGGCTACGTCCAGGAGCGCACC
ATCTTCTTCAAGGACGACGGCAACTACAAGACCCGCGCCGAGGTGAAGTTCGAGGGCGACACCCTGGTGAA
CCGCATCGAGCTGAAGGGCATCGACTTCAAGGAGGACGGCAACATCCTGGGGCACAAGCTGGAGTACAACT
ACAACAGCCACAACGTCTATATCATGGCCGACAAGCAGAAGAACGGCATCAAGGTGAACTTCAAGATCCGC
CACAACATCGAGGACGGCAGCGTGCAGCTCGCCGACCACTACCAGCAGAACACCCCCATCGGCGACGGCCC
CGTGCTGCTGCCCGACAACCACTACCTGAGCACCCAGTCCGCCCTGAGCAAAGACCCCAACGAGAAGCGCG
ATCACATGGTCCTGCTGGAGTTCGTGACCGCCGCCGGGATCACTCTCGGCATGGACGAGCTGTACAAGtaa

hsGLMP^{Y402A}$_{Gly\text{-}linker}$hsMFSD1^{AA}eGFP

ATGCGCGGCTCTGTGGAGTGCACCTGGGGTTGGGGGCACTGTGCCCCCAGCCCCCTGCTCCTTTGGACTCT
ACTTCTGTTTGCAGCCCCATTTGGCCTGCTGGGGGAGAAGACCCGCCAGGTGTCTCTGGAGGTCATCCCTA
ACTGGCTGGGCCCCCTGCAGAACCTGCTTCATATACGGGCAGTGGGCACCAATTCCACACTGCACTATGTG
TGGAGCAGCCTGGGGCCTCTGGCAGTGGTAATGGTGGCCACCAACACCCCCCACAGCACCCTGAGCGTCAA
CTGGAGCCTCCTGCTATCCCCTGAGCCCGATGGGGGCCTGATGGTGCTCCCTAAGGACAGCATTCAGTTTT
CTTCTGCCCTTGTTTTTACCAGGCTGCTTGAGTTTGACAGCACCAACGTGTCCGATACGGCAGCAAAGCCT
TTGGGAAGACCATATCCTCCATACTCCTTGGCCGATTTCTCTTGGAACAACATCACTGATTCATTGGATCC
TGCCACCCTGAGTGCCACATTTCAAGGCCACCCCATGAACGACCCTACCAGGACTTTTGCCAATGGCAGCC
TGGCCTTCAGGGTCCAGGCCTTTTCCAGGTCCAGCCGACCAGCCCAACCCCCTCGCCTCCTGCACACAGCA
GACACCTGTCAGCTAGAGGTGGCCCTGATTGGAGCCTCTCCCCGGGGAAACCGTTCCCTGTTTGGGCTGGA
GGTAGCCACATTGGGCCAGGGCCCTGACTGCCCCTCAATGCAGGAGCAGCACTCCATCGACGATGAATATG
CACCGGCCGTCTTCCAGTTGGACCAGCTACTGTGGGGCTCCCTCCCATCAGGCTTTGCACAGTGGCGACCA
GTGGCTTACTCCCAGAAGCCGGGGGGCCGAGAATCAGCCCTGCCCTGCCAAGCTTCCCCTCTTCATCCTGC
CTTAGCATACTCTCTTCCCCAGTCACCCATTGTCCGAGCCTTCTTTGGGTCCCAGAATAACTTCTGTGCCT
TCAATCTGACGTTCGGGGCTTCCACAGGCCCTGGCTATTGGGACCAACACTACCTCAGCTGGTCGATGCTC
CTGGGTGTGGGCTTCCCTCCAGTGGACGGCTTGTCCCCACTAGTCCTGGGCATCATGGCAGTGGCCCTGGG
TGCCCCAGGGCTCATGCTGCTAGGGGGCGGCTTGGTTCTGCTGCTGCACCACAAGAAGTACTCAGAGgcCC
AGTCCATAAATggtggaggttccggaggtggatcgggaggttctgccATGGAGGAGGAGGACGAGGAGGCA
CGGGCCgcagcaGCAGGAGGACCCGACGAGGCCGATAGAGGCGCCCCCGCCGCCCCTGGCGCCCTGCCTGC
CCTGTGCGATCCATCTCGGCTGGCCCACAGACTGCTGGTGCTGCTGCTGATGTGCTTCCTGGGCTTTGGCA
GCTACTTCTGTTATGACAACCCTGCCGCCCTGCAGACCCAGGTGAAGCGGGATATGCAAGTGAATACCACA
AAGTTTATGCTGCTGTACGCCTGGTATTCCTGGCCAAACGTGGTGCTGTGCTTCTTCGGCGGCTTCCTGAT
CGACAGGGTGTTTGGCATCCGCTGGGGCACAATCATCTTCTCTTGCTTCGTGTGCATCGGACAGGTGGTGT
TCGCACTGGGAGGCATCTTCAACGCCTTTTGGCTGATGGAGTTTGGCCGGTTCGTGTTTGGAATCGGAGGA
GAGAGCCTGGCAGTGGCACAGAACACCTACGCCGTGTCCTGGTTCAAGGGCAAGGAGCTGAATCTGGTGTT
TGGCCTGCAGCTGTCCATGGCCAGAATCGGCTCTACAGTGAACATGAATCTGATGGGCTGGCTGTATAGCA
AGATCGAGGCCCTGCTGGGATCCGCCGGACACACCACACTGGGAATCACCCTGATGATCGGCGGCATCACA
TGCATCCTGAGCCTGATCTGTGCCCTGGCCCTGGCCTACCTGGATCAGAGGGCCGAGCGCATCCTGCACAA
GGAGCAGGGCAAGACCGGCGAAGTGATCAAGCTGACAGACGTGAAGGATTTCTCCCTGCCACTGTGGCTGA
TCTTTATCATCTGCGTGTGCTACTATGTGGCCGTGTTCCCCTTTATCGGCCTGGGCAAGGTGTTCTTTACC
GAGAAGTTCGGCTTTAGCTCCCAGGCCGCCAGCGCCATCAATTCCGTGGTGTATGTGATCTCTGCCCCAAT
GAGCCCCGTGTTCGGCCTGCTGGTGGACAAGACAGGCAAGAACATCATCTGGGTGCTGTGCGCAGTGGCAG
CCACCCTGGTGAGCCACATGATGCTGGCCTTTACAATGTGGAATCCTTGGATCGCCATGTGCCTGCTGGGC
CTGTCTTACAGCCTGCTGGCATGTGCACTGTGGCCAATGGTGGCCTTCGTGGTCCCTGAGCACCAGCTGGG
AACCGCCTATGGCTTTATGCAGTCTATCCAGAACCTGGGCCTGGCCATCATCTCTATCATCGCCGGCATGA
TCCTGGATAGCAGGGGCTACCTGTTCCTGGAGGTGTTCTTTATCGCCTGCGTGAGCCTGTCTCTGCTGTCC
GTGGTGCTGCTGTACCTGGTGAATCGCGCCCAGGGCGGCAACCTGAATTATTCCGCCCGGCAGAGAGAGGA
GATCAAGTTCTCTCACACCGAGttaccggtcgccaccATGGTGAGCAAGGGCGAGGAGCTGTTCACCGGGG
TGGTGCCCATCCTGGTCGAGCTGGACGGCGACGTAAACGGCCACAAGTTCAGCGTGTCCGGCGAGGGCGAG
GGCGATGCCACCTACGGCAAGCTGACCCTGAAGTTCATCTGCACCACCGGCAAGCTGCCCGTGCCCTGGCC
CACCCTCGTGACCACCCTGACCTACGGCGTGCAGTGCTTCAGCCGCTACCCCGACCACATGAAGCAGCACG
ACTTCTTCAAGTCCGCCATGCCCGAAGGCTACGTCCAGGAGCGCACCATCTTCTTCAAGGACGACGGCAAC
TACAAGACCCGCGCCGAGGTGAAGTTCGAGGGCGACACCCTGGTGAACCGCATCGAGCTGAAGGGCATCGA
CTTCAAGGAGGACGGCAACATCCTGGGGCACAAGCTGGAGTACAACTACAACAGCCACAACGTCTATATCA
TGGCCGACAAGCAGAAGAACGGCATCAAGGTGAACTTCAAGATCCGCCACAACATCGAGGACGGCAGCGTG
CAGCTCGCCGACCACTACCAGCAGAACACCCCCATCGGCGACGGCCCCGTGCTGCTGCCCGACAACCACTA
CCTGAGCACCCAGTCCGCCCTGAGCAAAGACCCCAACGAGAAGCGCGATCACATGGTCCTGCTGGAGTTCG
TGACCGCCGCCGGGATCACTCTCGGCATGGACGAGCTGTACAAGtaa

hsMFSD1$_{2xmyc}$^{AA}eGFP

ATGGAGGAGGAGGACGAGGAGGCACGGGCCgcagcaGCAGGAGGACCCGACGAGGCCGATAGAGGCGCCCC
CGCCGCCCCTGGCGCCCTGCCTGCCCTGTGCGATCCATCTCGGCTGGCCCACAGACTGCTGGTGCTGCTGC
TGATGTGCTTCCTGGGCTTTGGCAGCTACTTCTGTTATGACAACCCTGCCGCCCTGCAGACCCAGGTGAAG
CGGGATATGCAAggtggaggctctGAACAGAAACTCATTAGCGAAGAGGACCTGGAGCAGAAACTCATCTC
AGAAGAAGACCTGggaggaggtgctGTGAATACCACAAAGTTTATGCTGCTGTACGCCTGGTATTCCTGGC
CAAACGTGGTGCTGTGCTTCTTCGGCGGCTTCCTGATCGACAGGGTGTTTGGCATCCGCTGGGGCACAATC
ATCTTCTCTTGCTTCGTGTGCATCGGACAGGTGGTGTTCGCACTGGGAGGCATCTTCAACGCCTTTTGGCT
GATGGAGTTTGGCCGGTTCGTGTTTGGAATCGGAGGAGAGAGCCTGGCAGTGGCACAGAACACCTACGCCG
TGTCCTGGTTCAAGGGCAAGGAGCTGAATCTGGTGTTTGGCCTGCAGCTGTCCATGGCCAGAATCGGCTCT
ACAGTGAACATGAATCTGATGGGCTGGCTGTATAGCAAGATCGAGGCCCTGCTGGGATCCGCCGGACACAC
CACACTGGGAATCACCCTGATGATCGGCGGCATCACATGCATCCTGAGCCTGATCTGTGCCCTGGCCCTGG
CCTACCTGGATCAGAGGGCCGAGCGCATCCTGCACAAGGAGCAGGGCAAGACCGGCGAAGTGATCAAGCTG
ACAGACGTGAAGGATTTCTCCCTGCCACTGTGGCTGATCTTTATCATCTGCGTGTGCTACTATGTGGCCGT
GTTCCCCTTTATCGGCCTGGGCAAGGTGTTCTTTACCGAGAAGTTCGGCTTTAGCTCCCAGGCCGCCAGCG
CCATCAATTCCGTGGTGTATGTGATCTCTGCCCCAATGAGCCCCGTGTTCGGCCTGCTGGTGGACAAGACA
GGCAAGAACATCATCTGGGTGCTGTGCGCAGTGGCAGCCACCCTGGTGAGCCACATGATGCTGGCCTTTAC
AATGTGGAATCCTTGGATCGCCATGTGCCTGCTGGGCCTGTCTTACAGCCTGCTGGCATGTGCACTGTGGC
CAATGGTGGCCTTCGTGGTCCCTGAGCACCAGCTGGGAACCGCCTATGGCTTTATGCAGTCTATCCAGAAC
CTGGGCCTGGCCATCATCTCTATCATCGCCGGCATGATCCTGGATAGCAGGGGCTACCTGTTCCTGGAGGT
GTTCTTTATCGCCTGCGTGAGCCTGTCTCTGCTGTCCGTGGTGCTGCTGTACCTGGTGAATCGCGCCCAGG
GCGGCAACCTGAATTATTCCGCCCGGCAGAGAGAGGAGATCAAGTTCTCTCACACCGAGttaccggtcgcc
accATGGTGAGCAAGGGCGAGGAGCTGTTCACCGGGGTGGTGCCCATCCTGGTCGAGCTGGACGGCGACGT
AAACGGCCACAAGTTCAGCGTGTCCGGCGAGGGCGAGGGCGATGCCACCTACGGCAAGCTGACCCTGAAGT
TCATCTGCACCACCGGCAAGCTGCCCGTGCCCTGGCCCACCCTCGTGACCACCCTGACCTACGGCGTGCAG
TGCTTCAGCCGCTACCCCGACCACATGAAGCAGCACGACTTCTTCAAGTCCGCCATGCCCGAAGGCTACGT
CCAGGAGCGCACCATCTTCTTCAAGGACGACGGCAACTACAAGACCCGCGCCGAGGTGAAGTTCGAGGGCG
ACACCCTGGTGAACCGCATCGAGCTGAAGGGCATCGACTTCAAGGAGGACGGCAACATCCTGGGGCACAAG
CTGGAGTACAACTACAACAGCCACAACGTCTATATCATGGCCGACAAGCAGAAGAACGGCATCAAGGTGAA
CTTCAAGATCCGCCACAACATCGAGGACGGCAGCGTGCAGCTCGCCGACCACTACCAGCAGAACACCCCCA
TCGGCGACGGCCCCGTGCTGCTGCCCGACAACCACTACCTGAGCACCCAGTCCGCCCTGAGCAAAGACCCC
AACGAGAAGCGCGATCACATGGTCCTGCTGGAGTTCGTGACCGCCGCCGGGATCACTCTCGGCATGGACGA
GCTGTACAAGtaa

hsGLMP^{Y402A}$_{Gly\text{-}linker}$hsMFSD1$_{2xmyc}$ ^{AA}eGFP

ATGCGCGGCTCTGTGGAGTGCACCTGGGGTTGGGGGCACTGTGCCCCCAGCCCCCTGCTCCTTTGGACTCT
ACTTCTGTTTGCAGCCCCATTTGGCCTGCTGGGGGAGAAGACCCGCCAGGTGTCTCTGGAGGTCATCCCTA
ACTGGCTGGGCCCCCTGCAGAACCTGCTTCATATACGGGCAGTGGGCACCAATTCCACACTGCACTATGTG
TGGAGCAGCCTGGGGCCTCTGGCAGTGGTAATGGTGGCCACCAACACCCCCCACAGCACCCTGAGCGTCAA
CTGGAGCCTCCTGCTATCCCCTGAGCCCGATGGGGGCCTGATGGTGCTCCCTAAGGACAGCATTCAGTTTT
CTTCTGCCCTTGTTTTTACCAGGCTGCTTGAGTTTGACAGCACCAACGTGTCCGATACGGCAGCAAAGCCT
TTGGGAAGACCATATCCTCCATACTCCTTGGCCGATTTCTCTTGGAACAACATCACTGATTCATTGGATCC
TGCCACCCTGAGTGCCACATTTCAAGGCCACCCCATGAACGACCCTACCAGGACTTTTGCCAATGGCAGCC
TGGCCTTCAGGGTCCAGGCCTTTTCCAGGTCCAGCCGACCAGCCCAACCCCCTCGCCTCCTGCACACAGCA
GACACCTGTCAGCTAGAGGTGGCCCTGATTGGAGCCTCTCCCCGGGGAAACCGTTCCCTGTTTGGGCTGGA
GGTAGCCACATTGGGCCAGGGCCCTGACTGCCCCTCAATGCAGGAGCAGCACTCCATCGACGATGAATATG
CACCGGCCGTCTTCCAGTTGGACCAGCTACTGTGGGGCTCCCTCCCATCAGGCTTTGCACAGTGGCGACCA
GTGGCTTACTCCCAGAAGCCGGGGGGCCGAGAATCAGCCCTGCCCTGCCAAGCTTCCCCTCTTCATCCTGC
CTTAGCATACTCTCTTCCCCAGTCACCCATTGTCCGAGCCTTCTTTGGGTCCCAGAATAACTTCTGTGCCT
TCAATCTGACGTTCGGGGCTTCCACAGGCCCTGGCTATTGGGACCAACACTACCTCAGCTGGTCGATGCTC
CTGGGTGTGGGCTTCCCTCCAGTGGACGGCTTGTCCCCACTAGTCCTGGGCATCATGGCAGTGGCCCTGGG
TGCCCCAGGGCTCATGCTGCTAGGGGGCGGCTTGGTTCTGCTGCTGCACCACAAGAAGTACTCAGAGgcCC
AGTCCATAAATggtggaggttccggaggtggatcgggaggttctgccATGGAGGAGGAGGACGAGGAGGCA
CGGGCCgcagcaGCAGGAGGACCCGACGAGGCCGATAGAGGCGCCCCCGCCGCCCCTGGCGCCCTGCCTGC
CCTGTGCGATCCATCTCGGCTGGCCCACAGACTGCTGGTGCTGCTGCTGATGTGCTTCCTGGGCTTTGGCA
GCTACTTCTGTTATGACAACCCTGCCGCCCTGCAGACCCAGGTGAAGCGGGATATGCAAggtggaggctct
GAACAGAAACTCATTAGCGAAGAGGACCTGGAGCAGAAACTCATCTCAGAAGAAGACCTGggaggaggtgc
tGTGAATACCACAAAGTTTATGCTGCTGTACGCCTGGTATTCCTGGCCAAACGTGGTGCTGTGCTTCTTCG
GCGGCTTCCTGATCGACAGGGTGTTTGGCATCCGCTGGGGCACAATCATCTTCTCTTGCTTCGTGTGCATC
GGACAGGTGGTGTTCGCACTGGGAGGCATCTTCAACGCCTTTTGGCTGATGGAGTTTGGCCGGTTCGTGTT
TGGAATCGGAGGAGAGAGCCTGGCAGTGGCACAGAACACCTACGCCGTGTCCTGGTTCAAGGGCAAGGAGC
TGAATCTGGTGTTTGGCCTGCAGCTGTCCATGGCCAGAATCGGCTCTACAGTGAACATGAATCTGATGGGC
TGGCTGTATAGCAAGATCGAGGCCCTGCTGGGATCCGCCGGACACACCACACTGGGAATCACCCTGATGAT
CGGCGGCATCACATGCATCCTGAGCCTGATCTGTGCCCTGGCCCTGGCCTACCTGGATCAGAGGGCCGAGC
GCATCCTGCACAAGGAGCAGGGCAAGACCGGCGAAGTGATCAAGCTGACAGACGTGAAGGATTTCTCCCTG
CCACTGTGGCTGATCTTTATCATCTGCGTGTGCTACTATGTGGCCGTGTTCCCCTTTATCGGCCTGGGCAA
GGTGTTCTTTACCGAGAAGTTCGGCTTTAGCTCCCAGGCCGCCAGCGCCATCAATTCCGTGGTGTATGTGA
TCTCTGCCCCAATGAGCCCCGTGTTCGGCCTGCTGGTGGACAAGACAGGCAAGAACATCATCTGGGTGCTG
TGCGCAGTGGCAGCCACCCTGGTGAGCCACATGATGCTGGCCTTTACAATGTGGAATCCTTGGATCGCCAT
GTGCCTGCTGGGCCTGTCTTACAGCCTGCTGGCATGTGCACTGTGGCCAATGGTGGCCTTCGTGGTCCCTG
AGCACCAGCTGGGAACCGCCTATGGCTTTATGCAGTCTATCCAGAACCTGGGCCTGGCCATCATCTCTATC
ATCGCCGGCATGATCCTGGATAGCAGGGGCTACCTGTTCCTGGAGGTGTTCTTTATCGCCTGCGTGAGCCT
GTCTCTGCTGTCCGTGGTGCTGCTGTACCTGGTGAATCGCGCCCAGGGCGGCAACCTGAATTATTCCGCCC
GGCAGAGAGAGGAGATCAAGTTCTCTCACACCGAGttaccggtcgccaccATGGTGAGCAAGGGCGAGGAG
CTGTTCACCGGGGTGGTGCCCATCCTGGTCGAGCTGGACGGCGACGTAAACGGCCACAAGTTCAGCGTGTC
CGGCGAGGGCGAGGGCGATGCCACCTACGGCAAGCTGACCCTGAAGTTCATCTGCACCACCGGCAAGCTGC
CCGTGCCCTGGCCCACCCTCGTGACCACCCTGACCTACGGCGTGCAGTGCTTCAGCCGCTACCCCGACCAC
ATGAAGCAGCACGACTTCTTCAAGTCCGCCATGCCCGAAGGCTACGTCCAGGAGCGCACCATCTTCTTCAA
GGACGACGGCAACTACAAGACCCGCGCCGAGGTGAAGTTCGAGGGCGACACCCTGGTGAACCGCATCGAGC
TGAAGGGCATCGACTTCAAGGAGGACGGCAACATCCTGGGGCACAAGCTGGAGTACAACTACAACAGCCAC
AACGTCTATATCATGGCCGACAAGCAGAAGAACGGCATCAAGGTGAACTTCAAGATCCGCCACAACATCGA
GGACGGCAGCGTGCAGCTCGCCGACCACTACCAGCAGAACACCCCCATCGGCGACGGCCCCGTGCTGCTGC
CCGACAACCACTACCTGAGCACCCAGTCCGCCCTGAGCAAAGACCCCAACGAGAAGCGCGATCACATGGTC
CTGCTGGAGTTCGTGACCGCCGCCGGGATCACTCTCGGCATGGACGAGCTGTACAAGtaa

Table S2. Protocol for generating knock-out cell lines.

**TUBE 1**		
21 µl	OptiMEM I	Thermo Fisher Scientific, #11058021
27 µl	Cas9 (1 µM)	IDT, Alt-R S.p. Cas9 Nuclease V3, #1081058
27 µl	cr/tracrRNA (1 µM)	IDT, Alt-R CRISPR-Cas9 tracrRNA
		IDT, Hs.Cas9.MFSD1.1.AB CRISPR-Cas9 crRNA
		IDT, Hs.Cas9.MFSD1.1.AE CRISPR-Cas9 crRNA
		IDT, Hs.Cas9.GLMP.1.AA CRISPR-Cas9 crRNA
mix by vortexing		
7.5 µl	Cas9 PLUS reagent	Invitrogen, #CMAX00001
mix by vortexing		
incubate for 5 min at 25°C		

**TUBE 2**		
75 µl	OptiMEM I	Thermo Fisher Scientific, #11058021
6 µl	CRISPRMAX	Invitrogen, #CMAX00001
mix		
add content of TUBE1		
incubate for 10 min at 25°C		
add 50 µl dropwise per 24-well		

Table S3. qPCR primer sequences used in this study.

	**5'-3'**
hsMFSD1_fw	ATGTGCTTCCTTGGCTTTG
hsMFSD1_rev	AACTGCTAAGGACTCGCCAC
hsGLMP_fw	CGCCAGGTGTCTCTGGA
hsGLMP_rev	GTTGGTGCTGTCAAACTCAA
hsGAPDH_fw	CAAGACCTTGGGCTGGGAC
hsGAPDH_rev	CTGTCGAACAGGAGGAGCAG

Table S4. Antibodies used in this study.

	**Supplier**	**Product Number**	
StrepMAB-Classic	IBA Lifesciences	2-1507-001	mouse monoclonal
anti-HA-tag, clone 3F10	Sigma	11867432001	rat monoclonal
anti-hs LAMP1 clone D2D11	Cell Signaling Technologies	9091T	rabbit monoclonal
anti-b-tubulin clone D3U1W	Cell Signaling Technologies	86298T	mouse monoclonal
anti-Cathepsin B D1C7Y	Cell Signaling Technologies	31718T	rabbit monoclonal
anti-LAMP2 clone H4B4	BioLegend	354302	mouse monoclonal
anti-myc tag 4A6	Sigma	05-724	mouse monoclonal
anti-GFP	E. Ogris lab (MPL, Vienna)		mouse monoclonal
anti-giantin	BioLegend	909701	rabbit polyclonal
anti-mouse IgG (H+L)-HRP	Bio-Rad	1721011	goat polyclonal
anti-rabbit IgG (H+L)-HRP	Bio-Rad	1706515	goat polyclonal
anti-rat IgG (H+L)-HRP	Thermo Fisher Scientific	31470	goat polyclonal
anti-rat Alexa Fluor 488	Thermo Fisher Scientific	A11006	goat polyclonal
anti-rabbit Alexa Fluor 555	Thermo Fisher Scientific	A31572	donkey polyclonal
anti-mouse Alexa Fluor 633	Thermo Fisher Scientific	A21050	goat polyclonal

Table S5. Dipeptides, tripeptides, and amino acids used in this study.

amino acids and dipeptides	Supplier	Product Number
H-Ala-Ala-OH	Bachem	4004429
H-Lys-Lys-OH	Bachem	4004666
H-Ala-Asp-OH	Bachem	4006259
H-Arg-Gly-Asp-OH	Bachem	4009173
H-Asp-Asp-OH	Bachem	4010210
H-Lys-Ala-OH	Bachem	4001389
H-Gly-His-Lys-OH	Bachem	4000308
H-Gly-Lys-Gly-OH	Bachem	4013188
H-Lys-Gly-OH	Bachem	4000317
H-Lys-Asp-OH	Bachem	4007825
H-Asp-Lys-OH	Bachem	4003468
H-Ala-Lys-OH	Bachem	4000977
H-Asp-Ala-OH	Bachem	4003132
H-Arg-Gly-OH	Bachem	4000541
H-His-Gly-OH	Bachem	4001227
Histidine	Carl Roth	1696.2
Lysine	Carl Roth	4207.2
Arginine	Carl Roth	1655.2
Aspartic acid	Sigma	A8949
Alanine	Sigma	29236

References

1. M. Abu-Remaileh *et al.*, Lysosomal metabolomics reveals V-ATPase- and mTOR-dependent regulation of amino acid efflux from lysosomes. *Science* **358**, 807-813 (2017).
2. K. L. Meerbrey *et al.*, The pINDUCER lentiviral toolkit for inducible RNA interference in vitro and in vivo. *Proc Natl Acad Sci U S A* **108**, 3665-3670 (2011)
